# Maternal thyroid function in the first half of pregnancy and neurodevelopmental outcomes in early adolescence in the Amsterdam Born Children and their Development (ABCD) cohort

**DOI:** 10.1016/j.cpnec.2025.100333

**Published:** 2025-12-22

**Authors:** Sarai M. Keestra, Susanne R. de Rooij, Tessa J. Roseboom, Marsh Königs, Tanja GM. Vrijkotte, Martijn J.J. Finken

**Affiliations:** a Department of Pediatric Endocrinology, Emma Children's Hospital, Amsterdam, the Netherlands; b Department of Epidemiology & Data Science, University of Amsterdam, Amsterdam, the Netherlands; c Amsterdam Reproduction & Development Research Institute, Amsterdam UMC, Amsterdam, the Netherlands; d Amsterdam Public Health Research Institute, Amsterdam UMC, Amsterdam, the Netherlands; e Emma Neuroscience Group, Emma Children's Hospital, Amsterdam, the Netherlands; f Amsterdam UMC, Location University of Amsterdam, Department of Public and Occupational Health, Amsterdam, the Netherlands

**Keywords:** Maternal thyroid function, Hypothyroxinaemia, Neurodevelopment, Longitudinal cohort studies, Amsterdam Born Children and their Development (ABCD) cohort

## Abstract

Maternal thyroid hormone levels in pregnancy have been implicated to play a role in brain development, but few observational studies have examined their relationship with neurodevelopmental outcomes beyond early childhood. We investigated associations between maternal thyroid function during the first 20 weeks of pregnancy and neurodevelopmental outcomes in 1824 children aged 10–13 years from the Amsterdam Born Children and their Development (ABCD) cohort. Outcomes included neurocognition (non-verbal intelligence [Raven's Progressive Matrices], executive working memory [SOPT]), behavioural organisation (behaviour regulation and metacognition [BRIEF], and internalizing behaviour and risk-seeking [SURPS]), and emotional, social, and behavioural problems (mother-, teacher-, and self-reported SDQ internalizing and externalizing problems). We fitted linear and quadratic multivariable regression models, as well as restricted cubic splines with three knots for the 10th, 50th and 90th percentiles, choosing the best model to assess associations across the continuous range of thyroid-stimulating hormone (TSH) and free thyroxine (FT4) on the outcomes, both in pregnancies without overt thyroid disease and in the full cohort. Secondary analyses examined potential threshold effects (10th and 90th percentiles of TSH and FT4), and impact of clinical thyroid dysfunction based on cohort-specific reference ranges, anti-TPO positivity, and sex differences. We found a small but significant positive linear association between maternal log-transformed TSH in pregnancies free of overt thyroid disease and non-verbal intelligence in girls (estimate: 0.05, 95 % CI: 0.01 to 0.09, p = 0.02; SMD: 0.015), but not in boys, after correction for multiple testing. No other neurodevelopmental outcomes were associated with maternal thyroid function. These findings suggest that maternal thyroid function in early pregnancy, in the absence of overt thyroid disease, shows no consistent associations with child neurodevelopmental outcomes in adolescence, apart from a small association with non-verbal intelligence in girls that may not be clinically relevant.

## Highlights

• We examined maternal thyroid function in early pregnancy in relation to adolescent neurodevelopment.
• Maternal TSH was positively associated with non-verbal IQ, but the effect size was small.
• This association was observed in girls only, not in boys.
• Maternal FT4 was not associated with adolescent neurodevelopment in pregnancies without overt thyroid disease.

## 1 Introduction

Thyroid hormones play an important role during the early stages of brain development, guiding dendrite proliferation, neuronal differentiation and migration, synapse formation, and myelination [[1], [2], [3]]. As the fetal thyroid gland does not gain its full capacity until around twenty weeks of gestation, the developing brain of the unborn child completely relies on the maternal thyroid function in early pregnancy, and continues to rely on adequate maternal intake of iodine, essential for thyroid hormone synthesis, throughout gestation [[4], [5], [6], [7], [8]]. Indeed, there is strong evidence from studies in both animals and humans that maternal iodine status and thyroid hormone sufficiency in gestation is important for the developing embryonic and fetal brain [[9], [10], [11], [12], [13], [14]].

Recent studies in population-based prospective cohorts suggest that even mild inadequacy of maternal thyroid hormone supply during early pregnancy can affect children's brain volumes, intelligence, and psychomotor development [[15], [16], [17], [18]]. Gestational thyroid hormone insufficiency has been associated with smaller grey matter and cortical volume at age 8 years [19], poorer expressive language functioning at 18 and 30 months as indicated by lower expressive vocabulary scores [20], and higher prevalence of symptoms of hyperactivity and attention deficit disorder in childhood [[21], [22], [23]]. This resonates with findings from a series of previous investigations in the Amsterdam Born Children and their Development (ABCD) cohort at age 5–6 years, showing that mild maternal hypothyroxinaemia during gestation, defined as being in the lowest 10th percentile of maternal thyroxine (FT4) levels, was associated with higher prevalence of teacher-reported hyperactivity and inattention problems [24], slower and more variable speed of information processing [25], as well as poorer performance on arithmetic tests but not language tests at school [26]. Large meta-analyses of individual participant data furthermore showed that higher maternal FT4 levels in early pregnancy are associated with adverse neurodevelopmental outcomes such as decreased intelligence, higher risk of attention-deficit hyperactivity disorder (ADHD) symptomatology, and a greater risk of autistic traits [19,27]. Together, these findings suggest that both lower and higher maternal thyroid hormone levels during pregnancy are associated with suboptimal neurodevelopment, consistent with an inverse U-shaped relationship between maternal thyroid function during pregnancy and children's neurodevelopmental outcomes [4].

Despite not directly affecting offspring neurodevelopment, maternal thyroid-stimulating hormone (TSH) reflects feedback regulation within the thyroid axis and may indicate insufficient thyroid hormone availability during pregnancy [28,29], whilst the presence of thyroid auto-antibodies, such as anti-TPO directed against thyroid peroxidase, signals autoimmune activity that can impair thyroid hormone synthesis, thereby reducing FT4 levels in pregnancy [28,30]. Both have been associated with children's neurodevelopmental outcomes after birth. Children from mothers with TSH levels that are high but still within the normal range had higher attention and hyperactivity/impulsivity symptoms, and score lower on quantitative, memory and verbal domains of the McCarthy development scale at the age of 4 years [31,32]. In accordance, a combination of high-normal TSH and low FT4, but not high-normal TSH and normal FT4, was associated with lower gross motor scores as infants [32], suggesting that the contextualisation of TSH levels in FT4 levels is important. To add a further layer of complexity, maternal anti-TPO positivity has been associated with reduced child IQ and higher risk of externalizing problems, particularly attention deficit/hyperactivity problems, even after correcting for TSH levels [33]. Together, these findings suggest that suboptimal thyroid hormone supply in pregnancy can affect neurocognitive, psychosocial, and behavioural outcomes after birth.

However, most studies on maternal thyroid function and child neurodevelopment have a limited follow-up time, focusing primarily on infants and preschool-aged children [20,[34], [35], [36], [37], [38]] and school-aged children [23,39,40]. Few studies have assessed long-term consequences of variation in maternal gestational thyroid function across the spectrum for neurodevelopment at adolescent age (i.e. 10-19 years). Furthermore, those that did focus on adolescents, have more often relied on clinical diagnoses of ADHD and school performance, both self-reported and through standardized test scores, than on extensive neurocognitive assessments. A Finnish study of children at age sixteen years found slightly lower scholastic performance based on self-rated mathematics performance as well as higher odds of having to repeat a class in children from mothers with thyroid function abnormalities during pregnancy [41]. On the other hand, British high schoolers born to mothers with either low or high thyroid hormone levels in pregnancy did not perform worse on their school assessments at age 15 than their peers [42]. Similarly, a recent study from Denmark involving more than 17,000 mother-child dyads did not find support for associations between maternal levels of TSH and FT4 during gestation and the odds of having a diagnosis for ADHD or autism, nor for lower school performance in late childhood and early adolescence [43]. Taken together, current evidence on adolescent outcomes is inconsistent, leaving open the question of whether maternal thyroid function during early pregnancy exerts lasting effects and underscoring the value of studies with broader neurodevelopmental assessments.

In this study we assessed neurodevelopment at ages 10–13 in the ABCD cohort, focusing on three domains; neurocognitive outcomes, behavioural organisation, as well as emotional, social and behavioural problems. These outcome domains were chosen to provide a more holistic assessment of adolescent functioning and to extend beyond the behavioural and neurocognitive aspects measured in the same cohort at age 5–6 years [[24], [25], [26]]. This broader scope allowed us to test whether maternal thyroid function in early pregnancy is linked not only to early childhood outcomes, but also to more complex neurocognitive and psychosocial domains in adolescence. Analysing this broader set of domains within one framework also reduces the risk of inflating associations through selective reporting, as correction for multiple testing can be applied consistently across outcomes. Given the limited evidence in this age group, our analyses were largely exploratory, with the expectation, based on earlier findings, that both lower and higher maternal FT4, as well as high-normal TSH or anti-TPO positivity, might be associated with variation across these domains.

## 2 Methods

### 2.1 Participants and recruitment

Pregnant women living in Amsterdam between January 2003 and March 2004 were eligible to participate in the Amsterdam Born Children and their Development cohort study (n = 12,393) and were recruited at the first prenatal visit (n = 8266) [44]. Next to completing a questionnaire, 4389 women gave informed consent for taking blood for a biomarker study during this prenatal visit, and for 4217 women valid measurements of thyroid function were obtained (TSH, FT4, and anti-TPO). When children from the ABCD cohort were between 10 and 13 years old, several indicators of psychosocial, behavioural and neurocognitive development were measured. Teachers, mothers, and children filled out questionnaires on psychosocial wellbeing, impulsivity and executive functioning. Using computerized tests, non-verbal intelligence and working memory were assessed in a smaller subset of the participating children. Overall, 1824 mother-child dyads with thyroid measurements and at least one neurodevelopmental outcome as described below were available. Medical ethical approval was obtained from the participating hospitals and the Registration Committee of the Health Municipality of Amsterdam. Mothers gave written informed consent for their children and written informed; consent was provided by children from 12 years old as well.

### 2.2 Inclusion and exclusion criteria

**Fig. 1:**
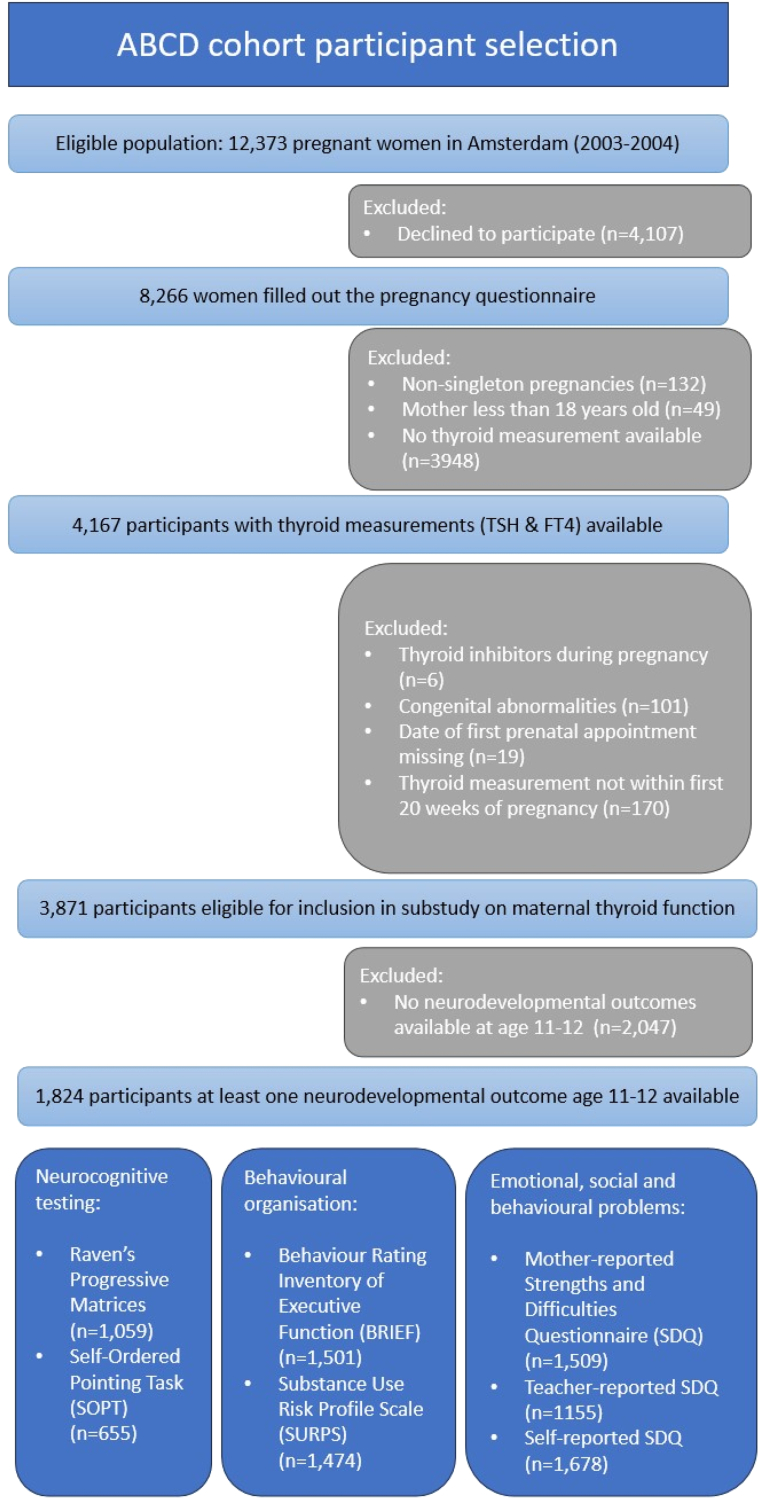
Flowchart of the selection of eligible participants from the ABCD Cohort.

Children whose mothers used thyroid inhibitors during pregnancy (n = 6) or with reported congenital malformations (n = 101) were excluded. We also excluded women for whom the gestational age at measurement was not available (n = 19) or for whom thyroid function was tested after 20 weeks of gestation (n = 170). Additionally, children without at least one measurement of neurodevelopment in the data collection wave that took place between the ages of 10.6–13.4 years (n = 2047) were excluded. This resulted in a final sample of 1824 mother-child dyads (Fig. 1). Women using levothyroxine during pregnancy were not excluded a priori, as unlike antithyroid drugs, levothyroxine is not associated with adverse pregnancy or birth outcomes [45]. To minimise potential confounding, these women were excluded from the primary analyses and included only in secondary sensitivity analyses.

### 2.3 Exposure: maternal thyroid function

TSH concentrations (manufacturer reference range for men and nonpregnant women: 0.34–5.60mU/L) and FT4 (manufacturer reference range for men and nonpregnant women: 7.5–21.1 pmol/L) were measured with access immunoanalyzer of Beckman Coulter Inc (Fullerton, California) in blood samples taken during the first prenatal visit [25]. Blood samples were collected between 8:05 and 18:00, with a median withdrawal time of 11:45. Sensitivity of the TSH assay was 0.1 mU/L, and the interassay coefficient of variation was 5.0 %. The lower limit of detection was 1.9 pmol/L for FT4, and the interassay coefficient of variation was 3.1 %–5.0 %. The cut-off for anti-TPO antibody positivity was >80kU/L and was determined by ELISA ELIZEN Tg Ab (E-CK- 96; ZenTech, Angleur, Belgium) with an interassay coefficient of variation of 13.4 %. In accordance with previous publications in the ABCD cohort [[24], [25], [26]], we standardized FT4 based on the median postmenstrual day (89) at which blood sampling occurred, based on the observation that it declined in a linear fashion (0.032 pmol/l per gestational day) in the first 20 weeks for FT4 but not TSH (Supplementary A). In the continuous analyses TSH was log-transformed to address skewness and approximate a normal distribution for statistical modelling. We then categorized the mother-child dyads into hypo-, eu- and hyperthyroxinaemic and hypo-, eu-, and hyperthyrotropinaemic based on the 10th and 90th percentiles for FT4 and TSH levels respectively. We differentiated between euthyroid mothers (TSH and FT4 in the trimester specific reference range for the ABCD cohort) and different clinical diagnoses of thyroid dysfunction, such as overt and subclinical hypo- and hyperthyroidism as well as clinical hypo- and hyperthyroxinaemia based on trimester-specific reference intervals of TSH and FT4 for the ABCD-cohort as developed by Osinga and colleagues [46] (Table 1). For anti-TPO positivity we used the manufacturer cut-off of 80 kU/l.

**Table 1:**
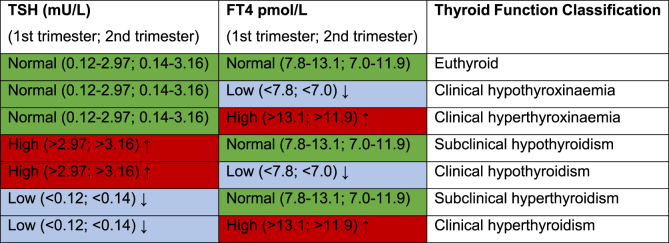
Thyroid dysfunction classified based on trimester-specific reference ranges for the first and second trimester for the ABCD cohort based on [46]

### 2.4 Neurodevelopmental outcomes

#### 2.4.1 Neurocognition

##### 2.4.1.1 Raven's Progressive Matrices for non-verbal intelligence

A subset of children in the ABCD study at participating schools were taken to a separate quiet room under supervision of a teacher to complete neurocognitive testing on a computer. Non-verbal intelligence was assessed using an adapted version of Raven's Progressive Matrices, a well-established paradigm for evaluating intelligence in children in primary school [47]. The Raven's Progressive Matrices consists of a series of visual puzzles which increase in difficulty as testing progresses and measures the logical reasoning and learning effect. An abbreviated version of 28 questions in twenty min was administered on a computer screen and the score is displayed as number of correct answers [48]. Only children with at least 13 valid responses were included to ensure reliable assessment, as excessive missing data could compromise the validity of individual scores. A total of 1059 children had valid measurements available.

##### 2.4.1.2 Self-ordered pointing test (SOPT)

Following the Raven's Progressive Matrices, participants completed the self-ordered pointing test (SOPT), a computerized task for measuring working memory, executive function, and speed [49,50]. After a first practice round, the children completed six rounds in 10 min, in which a child got 4,8, or 12 images, on which the child was only supposed to click once. After each click, the images were shuffled, and each image had to be clicked at least once but not more. Total error score reflecting the proportion of wrong answers has acceptable reliability [49]. A total of 655 children had valid measurements available. The reason that fewer children had valid measurements for the SOPT than for the Raven's Progressive Matrices is due to technical difficulties with the loading of the test at schools with an unstable internet connection, due to which some children who completed the Raven's Progressive Matrices were not able to complete the SOPT.

#### 2.4.2 Behavioural organization and risk-taking

##### 2.4.2.1 Behaviour Rating Inventory of Executive Function (BRIEF)

An abbreviated version of the Behaviour Rating Inventory of Executive Function (BRIEF) [51], a questionnaire evaluating eight aspects of executive functioning in real-world settings, was administered to the mothers (n = 1501). The BRIEF can be subdivided in two indices; the Behavioural Regulation Index (BRI) combining Inhibit, Shift, and Emotional Control, which assesses children's ability to display emotional reactions appropriate for the context and developmental stage; and the Metacognition Index combining Initiate, Working Memory, Plan/Organize, Organization of Materials and Monitor scales reflecting the ability of a child to cognitively manage tasks adequately through goal-oriented behaviours and effective initiation, planning, organising and monitoring of these behaviours [34,52]. For the BRIEF, good construct validity and excellent intra-rater reliability have been reported, whereas inter-rater reliability is not expected in a measurement tool that evaluates context specific behaviours (i.e. a child might behave differently at home than in the classroom) [52].

##### 2.4.2.2 Substance Use Risk Profile Scale (SURPS)

Children (n = 1474) completed an age-adjusted version of the Substance Use Risk Profile Scale (SURPS) [53], which measures anxiety sensitivity and hopelessness to assess internalizing traits, as well as sensation seeking and impulsivity to assess risk-taking behaviour [54]. The SURPS has high validity and acceptable test-retest reliability [53].

#### 2.4.3 Emotional, social and behavioural problems

##### 2.4.3.1 Strengths and Difficulties Questionnaire (SDQ)

Finally, mothers (n = 1509), teachers (n = 1155), and children themselves (n = 1678) completed the Dutch version of the Strengths and Difficulties Questionnaire (SDQ) [55,56]. The SDQ is a widely used screening tool consisting of 25 questions assessing symptoms of behavioural and emotional disorders in children [56]. The scales of the SDQ can be recombined to assess Internalizing Problems (Emotional Symptoms and Peer Problems) as well as Externalizing Problems (Hyperactivity/Inattention and Conduct Problems) [57].

### 2.5 Covariate selection

**Fig. 2:**
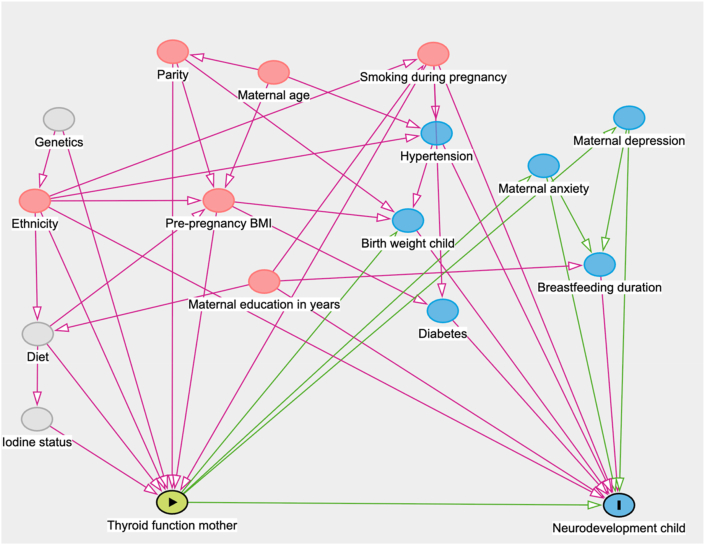
Directed acyclic graph (DAG) depicting hypothesized causal relations between maternal thyroid function, child neurodevelopment, and potential confounders.

Based on the covariates used in earlier publications on the neurodevelopmental outcomes of children in the ABCD cohort age 5–6 years [[24], [25], [26]] and a review of relevant literature, we created a causal model represented in a directed acyclic graph (DAG) using *dagitty* software [58]. A DAG is a diagram that summarises assumed causal relationships between exposure, outcome, and potential confounders. Nodes represent variables and arrows represent hypothesized directions of causal influence. By mapping these relationships before analysis, DAGs help identify which variables need to be adjusted for to obtain unbiased estimates of the association of interest, applying the backdoor criterion to block all non-causal pathways[[59], [60], [61]]. For transparency and reproducibility, the full graph is shown in Fig. 2. The DAG also visualises unmeasured or latent pathways (shown as grey nodes) highlighting possible sources of residual confounding and supporting our choice of a minimally sufficient adjustment set. The DAG indicated adjustment for maternal age during pregnancy (years), ethnicity (Dutch/non-Dutch), maternal education (years after primary school), parity (number of deliveries prior to current pregnancy), maternal pre-pregnancy BMI (kg/m^2^), and maternal smoking during pregnancy (any vs none). Additionally, we adjusted all analyses for the age of assessment and sex of the child unless otherwise specified. After enrolment, mothers were given questionnaires that included age, ethnicity, parity, years of education, smoking during pregnancy and medication use. Pre-pregnancy body mass index (BMI) was calculated based on self-reported weight and height before pregnancy. Sex of the child was recorded at birth. Any missing covariates (<15 %) were imputed using the Multivariate Imputation by Chained Equations in R (mice) package with predictive mean matching (5 imputations, 10 iterations), averaging imputations for numeric variables (mean) and categorical variables (mode) (Supplementary A) [62].

### 2.6 Statistical analyses

#### 2.6.1 Primary analysis

All analyses were conducted in R studio version 4.2.1 using packages “rms” [63], “mgcv"[64], and “betareg"[65]. The goal of the primary analysis was to assess the association between maternal thyroid function (i.e. continuous levels of TSH and FT4) in relation to neurodevelopmental outcomes in mother-child dyads, first evaluating pregnancies free of thyroid disease based on the cohort specific reference values (Table 1). From the 1824 mother-child pairs, we selected a healthy subsample without maternal hypo- or hyperthyroidism and without use of thyroid hormone replacement therapy during pregnancy as well as with children ≥ 32 weeks gestation and ≥ 1,500g weight at birth (n = 1774). The reason for excluding very preterm and very low birth weight children in the initial analysis is because children in this group may suffer from transient hypothyroxinaemia of prematurity, which can affect postnatal thyroid function and neurodevelopment independent of maternal thyroid function in early gestation [66]. In accordance with previous publications [19,27], we first fitted three model types to evaluate associations between standardized FT4 and log-TSH and neurodevelopmental outcomes: linear models, quadratic models, and restricted cubic splines (RCS) with knots at the 10th, 50th, and 90th percentiles. Model fit was compared using the Akaike Information Criterion (AIC), and linear models provided the best fit compared to quadratic or RCS models. After running the linear regression models adjusting for the selected covariates, we display the original p-value for transparency, but only report results as significant in the main text if p < 0.05 after we applied False-Discovery Rate (FDR) correction using the Benjamini-Hochberg method at the group domain level of the outcomes (i.e. in the three domains of the outcomes neurocognitive outcomes (Raven's Progressive Matrices & SOPT), behavioural organisation (BRIEF & SURPS), and emotional, social and behavioural problems (SDQ as rated by teachers, mothers or children themselves)). For outcomes where TSH showed a significant association, we tested an interaction term between FT4 and TSH to assess whether the effect was moderated by FT4, as it is the biologically active thyroid hormone that crosses the placenta. For models with statistically significant associations, we quantified the effect size using Cohen's *d* for linear regression models of continuous outcomes and the “countES” R package (https://github.com/stefanycoxe/countES) for negative binomial or Poisson regressions to compute the standardized mean difference (SMD; an analogue of Cohen's d), with 95 % Monte Carlo confidence intervals [67]. We used marginal predictions from each fully adjusted model to estimate expected outcome scores at the mean and at one standard deviation above the mean of the standardized exposure (i.e., *z* = 0 and *z* = 1), fixing covariates at sample means or reference levels for categorical variables, to quantify expected absolute and relative differences. For log-transformed TSH, this corresponded to log(TSH) values of μ = X and μ + σ = Y, which we back-transformed to raw TSH values using exp(μ) and exp(μ + σ).

#### 2.6.2 Sensitivity analysis

We repeated the models in the primary analyses after reincluding mothers with children who were born very preterm (i.e. ≤32 weeks of gestation) or at very low birth weights (≤1500g) and mothers who suffered from overt hypo- or hyperthyroidism during pregnancy or who were on thyroid hormone supplements at their first prenatal visit [[24], [25], [26]], again applying FDR correction at the domain level.

#### 2.6.3 Secondary analyses

##### 2.6.3.1 Percentile cut-offs in the continuous distribution of TSH and FT4

As exploratory analyses we investigated the existence of a threshold in thyroid function for affecting neurodevelopmental outcomes by comparing within the population distribution FT4 euthyroxinaemic mother-child dyads with hypo- and hyperthyroxinaemic dyads (FT4 in 10th or 90th percentile respectively), and for TSH euthyrotropinaemic mother-child dyads with hypo- and hyperthyrotropinaemic dyads (TSH in 10th or 90th percentile respectively). For this analysis all mother-child dyads were included.

##### 2.6.3.2 Sex-specific effects and sex-differences

To investigate biologically plausible sex differences (i.e., whether FT4 and TSH influence outcomes in both sexes but to different degrees) or sex-specific effects (i.e., whether FT4 and TSH are associated with outcomes in only one sex but not the other) [68,69], we included interaction terms for FT4 and TSH with sex in the primary analyses. Sex-stratified analyses were performed when the interaction term reached *p* < 0.10, a threshold chosen to account for the lower statistical power of interaction tests and to avoid overlooking potential effect modification in this exploratory analysis [70].

##### 2.6.3.3 Clinical thyroid dysfunction and Anti-TPO positivity

Finally, we differentiated between euthyroid mothers (TSH and FT4 in the trimester specific reference range for the ABCD cohort) and different clinical diagnoses of thyroid dysfunction, such as overt and subclinical hypo- and hyperthyroidism as well as clinical hypo- and hyperthyroxinaemia based on trimester-specific reference intervals of TSH and FT4 for the ABCD-cohort as developed by Osinga and colleagues [46] (Table 1), and grouped mothers based on anti-TPO positivity based on manufacturer cut-off of 80 kU/l. For these exploratory analyses, we did not apply FDR correction.

## 3 Results

### 3.1 Primary analysis

#### 3.1.1 Continuous evaluation of TSH and FT4 levels

Compared to the original mother-child dyads of the ABCD cohort whose thyroid function was tested during pregnancy, the mothers of the children in the follow-up cohort on average were older, more highly educated, more often of Dutch ethnicity, and less likely to smoke during pregnancy, and their children had a slightly higher birth weight and gestational age (Supplementary A). The primary continuous analyses contained 1774 mother-child dyads free of overt thyroid disease excluding children born very prematurely (≤32 weeks) or at very low birth weights (≤1500g) (Table 2). Based on AIC comparisons, linear models using standardised FT4 and log-TSH provided the best fit. Including quadratic terms or restricted cubic splines did not improve model fit. After adjustment for potential confounders, higher maternal TSH was associated with higher non-verbal intelligence scores in offspring (estimate: 0.04, 95 % CI: 0.02 to 0.06, p = 0.03; Table 3). We modelled standardised log-transformed TSH and used marginal predictions at the mean and at one standard deviation above the mean of the exposure. These correspond to raw TSH values of 0.52 and 2.18 mIU/L. The predicted Raven score increased from 15.59 to 16.15 points (Δ = 0.56), a 3.6 % increase. This corresponds to a standardised mean difference of 0.010 (95 % CI: 0.003 to 0.018), indicating a small effect. There were no other significant associations between maternal thyroid function during pregnancy within the clinical reference range and the neurodevelopmental outcomes of their offspring. The interaction term between TSH and FT4 for non-verbal intelligence was not significant (p = 0.42).

**Table 2:** Characteristics of the mother-child dyads of the ABCD Cohort.

Variable	Entire sample	Hypo-thyroxinaemia (lowest 10th percentile FT4)	Euthyroxinaemia (middle 10th to 90th percentile FT4)	Hyper-thyroxinaemia (highest 90th percentile FT4)
Number of Mother-Child Dyads	1824	183	1458	183
Girls	944 (51.75 %)	97 (53.01 %)	760 (52.13 %)	87 (47.54 %)
Age of Child in Years At Testing in Years	11.56 (0.31)	11.61 (0.33)	11.56 (0.31)	11.54 (0.3)
Maternal Age During Pregnancy in Years	32.03 (4.07)	32.08 (4.15)	32.02 (4.11)	32.09 (3.72)
Parity	0.52 (0.75)	0.62 (0.88)	0.51 (0.74)	0.55 (0.7)
Dutch Ethnicity	1334 (73.14 %)	137 (74.86 %)	1071 (73.46 %)	126 (68.85 %)
Maternal Education in Years	10.29 (3.4)	9.56 (3.53)	10.37 (3.37)	10.39 (3.45)
Smoking During Pregnancy	121 (6.63 %)	23 (12.57 %)	89 (6.1 %)	9 (4.92 %)
Pre-Pregnancy BMI in kg/m^2^	22.67 (3.42)	23.85 (4.17)	22.57 (3.31)	22.26 (3.26)
Birth Weight Child in Grams	3508 (544)	3534 (639)	3507 (531)	3484 (540)
Thyroid Testing Gestational Week	12.9 (2.1)	12.7 (2.0)	13 (2.1)	12.6 (2.4)
FT4 (pmol/L)	9.74 (1.56)	7.78 (0.71)	9.64 (0.9)	12.51 (2.42)
TSH (mU/L)	1.39 (1.71)	2.5 (4.62)	1.3 (0.85)	0.93 (0.73)
Anti-TPO Positive	114 (6.25 %)	37 (20.22 %)	66 (4.53 %)	11 (6.01 %)

^a^ Variables are given as means (SD) except where given as percentages, when absolute number (%) is described.

**Table 3:** Associations between maternal thyroid function (TSH and FT4) during the first twenty weeks of gestation and neurodevelopmental outcomes of the child using regression models.

Neurodevelopmental outcome	Unadjusted models				Adjusted models^c^					
	Thyroid Parameter^a^	Estimate	Standard error	*p*-value	Estimate	Standard error	*p*-value	*p*-value after FDR correction^b^	Confidence interval lower bound	Confidence interval higher bound
Non-verbal intelligence	FT4	−0.02	0.01	0.10	−0.02	0.01	0.05	0.11	−0.04	0.00
Non-verbal intelligence	**TSH**	**0.04**	**0.01**	**0.00**	**0.04**	**0.01**	**0.01**	**0.03**	**0.02**	**0.06**
Executive working memory	FT4	−0.03	0.03	0.30	−0.01	0.03	0.65	0.68	−0.07	0.05
Executive working memory	TSH	−0.02	0.03	0.48	−0.01	0.03	0.68	0.68	−0.07	0.05
Behavioural regulation	FT4	0.00	0.01	0.57	0.00	0.01	0.53	0.90	−0.02	0.02
Behavioural regulation	TSH	0.00	0.01	0.73	0.00	0.01	0.56	0.90	−0.02	0.02
Metacognition	FT4	0.00	0.01	0.77	0.00	0.01	0.55	0.90	−0.02	0.02
Metacognition	TSH	0.00	0.01	0.64	0.00	0.01	0.84	0.96	−0.02	0.02
Internalizing traits	FT4	−0.02	0.01	0.08	−0.01	0.01	0.28	0.90	−0.03	0.01
Internalizing traits	TSH	−0.02	0.01	0.06	−0.02	0.01	0.13	0.90	−0.04	0.00
Risk taking behaviour	FT4	0.00	0.01	0.79	0.00	0.01	0.82	0.96	−0.02	0.02
Risk taking behaviour	TSH	0.00	0.01	0.81	0.00	0.01	1.00	1.00	−0.02	0.02
Mother-Reported Externalizing Problems	FT4	−0.02	0.02	0.35	−0.02	0.02	0.52	0.75	−0.06	0.02
Mother-Reported Externalizing Problems	TSH	0.01	0.03	0.79	0.01	0.03	0.69	0.75	−0.05	0.07
Mother-Reported Internalizing Problems	FT4	−0.05	0.03	0.04	−0.04	0.03	0.17	0.69	−0.10	0.02
Mother-Reported Internalizing Problems	TSH	0.01	0.03	0.68	0.02	0.03	0.39	0.75	−0.04	0.08
Teacher-Reported Externalizing Problems	FT4	−0.04	0.04	0.29	−0.04	0.04	0.32	0.75	−0.12	0.04
Teacher-Reported Externalizing Problems	TSH	−0.06	0.04	0.20	−0.02	0.04	0.64	0.75	−0.10	0.06
Teacher-Reported Internalizing Problems	FT4	−0.10	0.04	0.01	−0.08	0.04	0.03	0.19	−0.16	0.00
Teacher-Reported Internalizing Problems	TSH	−0.02	0.04	0.55	0.00	0.04	0.95	0.95	−0.08	0.08
Self-Reported Externalizing Problems	FT4	−0.01	0.02	0.42	−0.01	0.02	0.65	0.75	−0.05	0.03
Self-Reported Externalizing Problems	TSH	−0.01	0.02	0.74	−0.01	0.02	0.53	0.75	−0.05	0.03
Self-Reported Internalizing Problems	FT4	−0.08	0.02	0.00	−0.06	0.02	0.01	0.13	−0.10	−0.02
Self-Reported Internalizing Problems	TSH	0.01	0.02	0.77	0.01	0.02	0.56	0.75	−0.03	0.05

a FT4 was standardized for the median gestational day of testing (89 days) and TSH was log-transformed, both variables were scaled before analysis.

b False-Discovery Rate correction was applied at the outcome level of neurocognition, behavioural organization, and emotional, social, and behavioural problems respectively.

c All adjusted models were adjusted for the following covariates maternal age, pre-pregnancy-BMI, ethnicity, maternal education in years, parity prior to current pregnancy and maternal smoking during pregnancy.

#### 3.1.2 Sensitivity analyses

When including all mother-child dyads (n = 1824), also those with overt thyroid disease, children that were born preterm, low birth weight, or from mothers taking thyroid hormone replacement therapy at the first antenatal appointment, this did not change our findings from the primary analysis (Supplementary B). Higher TSH levels remained significantly associated with non-verbal intelligence scores (estimate: 0.03, 95 % CI: 0.004 to 0.057; p = 0.03). The sensitivity model yielded a similar effect size (SMD = 0.010, 95 % CI: 0.002–0.018). The interaction term between TSH and FT4 for non-verbal intelligence was not significant (p = 0.14).

### 3.2 Secondary analyses

#### 3.2.1 Percentile cut-offs in the continuous distribution of TSH and FT4

Including all mother-child dyads, we looked at differences in neurodevelopmental outcomes based on being in the lowest 10th percentile for FT4 (<8.28 pmol/L) or highest 90th percentile for FT4 (>11.34 pmol/L) or the lowest 10th percentile for TSH (<0.45 mU/l) or highest 90th percentile for TSH (>2.28 mU/l) of the distribution compared to the middle 10th-90th percentile (Supplementary C). There were no significant associations after correction for multiple testing.

#### 3.2.2 Sex-specific effects and sex-differences

The interaction term analysis suggested the existence of sex-differences for the association between TSH and non-verbal intelligence (p = 0.05), TSH as well as FT4 and mother-reported externalizing problems (p = 0.05 and p = 0.03 respectively), and TSH and self-reported externalizing and internalizing problems and TSH (p = 0.04 and p = 0.07) (Supplementary D). After sex stratification and FDR correction, we found a significant association in girls, but not in boys, between higher maternal TSH and higher non-verbal intelligence (estimate: 0.05, 95 % CI: 0.01–0.09; p = 0.02; Supplementary E and F). In girls, a one standard deviation increase in TSH (0.50–2.19 mIU/L), derived by back-transforming the mean ± 1 SD of log-transformed TSH, was associated with a model-predicted increase of 0.77 Raven points (from 15.32 to 16.10; +5.1 %), corresponding to a standardised mean difference of 0.015 (95 % CI: 0.005–0.025). No other associations were significant in the stratified models.

#### 3.2.3 Clinical thyroid dysfunction and Anti-TPO positivity

We evaluated the neurodevelopmental outcomes of children from mothers with overt hypothyroidism (n = 13), overt hyperthyroidism (n = 12), subclinical hypothyroidism (n = 56), subclinical hyperthyroidism (n = 31), clinical hypothyroxinaemia (n = 36) and clinical hyperthyroxinaemia (n = 28) compared to mother-child dyads from euthyroid pregnancies (n = 1649). Due to the small number of participants in each subgroup, we did not apply FDR correction in this exploratory analysis to avoid inflating the risk of type II errors. In children from mothers with subclinical hyperthyroidism we found lower mother-reported externalizing problems (estimate: 0.55; 95 % CI -0.90 to −0.03) and in children from mothers with clinical hyperthyroxinaemia lower internalizing problems (estimate: 0.48; 95 % CI: 0.83 to −0.03) compared to euthyroid mother-child dyads (Supplementary G). Finally, we compared the anti-TPO positive women (n = 114) with the women that were negative for this auto-antibody (n = 1710) and found no significant differences in the neurodevelopmental outcomes of their children. (Supplementary H).

## 4 Discussion

In this evaluation of the association between maternal thyroid function in the first half of pregnancy with children's neurodevelopmental outcomes in early adolescence, we found that non-verbal intelligence was slightly higher when TSH was higher; an association that was only observed in girls but not boys after sex-stratification. In girls this finding remained significant after FDR correction but the effect size was small, suggesting limited clinical significance. FT4 showed no independent association with non-verbal intelligence and did not modify the TSH–intelligence relationship, as interaction terms between FT4 and TSH were not significant. The other neurodevelopmental outcomes did not show a significant association with maternal thyroid function during pregnancy when children are at an early adolescent age. These findings indicate a small, sex-specific association between higher maternal TSH and slightly higher non-verbal intelligence in girls, while no significant associations were identified for other neurodevelopmental outcomes.

Given that an earlier study from the ABCD cohort reported increased odds of subnormal arithmetic performance at age 5–6 in children from pregnancies in the lowest 10th percentile of standardized FT4 [26], a potential indicator of non-verbal intelligence, we expected that children in the lowest 10th percentile of FT4 at an early adolescent age would also show lower non-verbal intelligence. A meta-analysis of intelligence scores in primary school-aged children from three cohorts similarly found that being born to mothers with FT4 levels below the 2.5th or 5th percentile was associated with lower non-verbal intelligence [27]. However, no significant association was found between FT4 and non-verbal intelligence in our study when children were between ten and thirteen years old. One possible explanation for the discrepancy is that these earlier studies assessed neurocognitive outcomes in early childhood, when the effects of low maternal FT4 may be more pronounced, whereas our study examined neurodevelopment in early adolescence, by which time early deficits may have been outgrown or mitigated through environmental or developmental factors.

We did, however, observe a positive association between maternal TSH and non-verbal intelligence in our cohort that was small but statistically significant. Levie et al. [27] reported a nonsignificant trend toward higher non-verbal IQ in children of mothers within the highest percentiles of TSH, which may point to a subtle pattern that is difficult to detect consistently across cohorts. Unlike our study, Levie et al. [27] included women with clinically abnormal thyroid function in their main analyses, which may account for the difference in findings, although in our study the finding of a small positive association persisted after a sensitivity analysis in which we included women with thyroid dysfunction during the first 20 weeks of pregnancy in a cohort free of overt thyroid disease. The finding that higher TSH is associated with higher non-verbal intelligence seems paradoxical, as high TSH is typically seen as a marker for thyroid insufficiency in pregnancy [71], and TSH is normally suppressed in early gestation by high human chorionic gonadotropin (hCG) concentrations. However, it could be speculated that in some women, a modestly higher TSH despite normal FT4 could perhaps represent mild iodine-related stimulation rather than incipient subclinical or overt hypothyroidism perse. In such cases, mildly elevated TSH, especially when accompanied by normal FT4, could represent a compensatory upregulation aimed at maintaining adequate hormone availability during early gestation. This interpretation is supported by Hoermann et al. [72], who argued that in euthyroid, non-pregnant individuals, moderately elevated TSH may reflect successful homeostatic adaptation under system stress rather than dysfunction. Chatzitomaris et al. [73] conceptualized this dynamic adjustment of set points in response to physiological stressors, such as pregnancy, as thyroid allostasis type 2, in which energy stores are mobilized to meet anticipated demands. Within this framework, it could be hypothesized that the observed weak positive association may indicate effective maternal adaptation that supports, rather than impairs, fetal neurodevelopment, particularly in a population with generally sufficient but heterogeneous iodine intake. Although the Netherlands is generally considered iodine sufficient [74,75], public health authorities have estimated that the diet of around one in ten pregnant women is iodine deficient, creating a context in which such compensation may be relevant. Zimmermann (2015) describes how, in moderate iodine deficiency and non-pregnant individuals, TSH may rise while FT4 remains stable due to preferential T3 secretion. Whether similar compensatory shifts occur in early pregnancy, when hCG suppresses TSH but iodine demand is increased, remains to be established.

A noteworthy observation from our study is that the association of TSH with non-verbal intelligence was present in girls but not in boys after sex-stratification. This raises the question as to whether variation in maternal thyroid function during gestation might have different associations with offspring neurodevelopmental outcomes based on the sex of the offspring. For example, in a study of mother-child dyads in Finland at the age of 8, researchers found that higher levels of maternal TSH were associated with higher levels of ADHD symptoms in girls but not in boys [76], but similar studies have not further investigated the existence of sex-specific effects in the associations they reported [22,23,76]. Earlier studies on maternal thyroid hormone exposure in relation to intelligence outcomes have generally not explicitly considered the role of sex in separate analyses [10,15,27,38]. The question also remains through what hypothesized biological pathway the brain of the female offspring would be affected differently than that of the male. The more widespread study of sex-specific neurodevelopmental effects in this context may shed further light on whether maternal thyroid abnormalities in pregnancy affect offspring differently based on their biological sex.

In an earlier assessment of the neurodevelopmental outcomes of 5-6-year-olds in the same cohort, it was found that children from mothers within the lowest 10th percentile of standardized FT4 values had higher odds of teacher-reported hyperactivity/inattention on the SDQ [24]. Yet in this study we did not find higher teacher-reported externalizing problems in the lowest 10th percentile group of FT4 values or in the clinical hypothyroxinaemia group. In a meta-analysis of individual participant data of nearly eight thousand mother-child dyads, a higher maternal FT4 level was associated with an increased risk of the offspring having ADHD symptoms above 90th percentile [77], but we did not find that higher FT4 was associated with higher scores of mother-, teacher-, or self-reported externalizing score. Instead, we observed a trend suggesting fewer internalizing problems in children of mothers with the highest 90th percentile of FT4, though this association did not persist after correction for multiple testing. While this may reflect a chance finding, further research using longitudinal data and population-specific SDQ cut-offs is needed to determine its clinical relevance.

### 4.1 Strengths and limitations

There are several noteworthy limitations to our study. Firstly, although our sample was representative of the pregnant population in Amsterdam at the time, the cohort was not large enough to investigate the impact of clinical thyroid disorders perse, as only very few of the pregnant women in the ABCD cohort could really be considered thyroxine deficient or suffering from excess according to trimester- and cohort specific reference ranges [46]. Therefore, larger sample sizes are needed from cross-cohort analyses to establish neurodevelopmental differences for clinical thyroid disorders in particular. However, we did find associations between maternal thyroid function in gestation and neurodevelopmental outcomes in the general population, even when assessing only mother-child dyads free of overt thyroid disease excluding very premature or very low weight births. It is worth noting that the follow-up sample differed from the original cohort: mothers were older, more often Dutch, higher educated, and less likely to smoke, and their children had slightly higher birth weights. This likely reflects selection toward a healthier, more advantaged subgroup, which may have reduced variation in both exposure and outcome, attenuating observed associations. Secondly, it could be that some of the neurocognitive assessment tools or behavioural questionnaires that we used are not sensitive enough for measuring the type of alterations that can be expected in children from pregnancies with differential gestational thyroid hormone exposure in the first trimester. However, many of these have been in use for both clinical and research purposes for decades such as the Raven Progressive Matrices and the Strength and Difficulties Questionnaires, and have well established paradigms [47,78], and others were specifically aimed at the impulsivity and task planning problems that one would expect to arise in children with higher than expected hyperactivity/inattention symptoms as was reported by teachers at age 5–6 years [24]. It is worth noting here that due to technical difficulties with the internet connection at some of the schools, the SOPT was only available for some, but not all of the children that completed the Raven's Progressive Matrices, which lead to a substantial smaller sample size for this neurodevelopmental outcome than for the other indicators. Thirdly, we did not have thyroid function measurements for the children available, and although we did exclude children with congenital abnormalities, including congenital hypothyroidism, we could not exclude children with thyroid dysfunction at the time of the assessment. Finally, it could be that previous studies, which have found associations of maternal thyroid function, particularly hypothyroxinaemia, at a pre-school or early school-age, tested at a time when these differences were still relevant, whereas after several years in the learning system, they become of less importance and other environmental and developmental factors may exert a stronger influence on neurocognition and behaviour.

### 4.2 Implications for clinical practice and future research

Despite widespread implementation of gestational thyroid screening and supplementation, current evidence shows limited benefit for offspring, possibly due to late testing (end of first trimester) when key neurodevelopment has already occurred [17,79,80]. Furthermore, most association studies evaluate children's developmental outcomes at a very early age, at which children might still outgrow any deficits during further development. Long-term follow-up of transient hypothyroxinaemia of prematurity, i.e., a transient reduction in FT4 levels occurring immediately after preterm birth, suggests early deficits in intelligence and motor function development did not persist into adolescence when adjusting for demographic factors [81]. In our study, previously reported associations with externalizing problems were not significant after correction for multiple testing at a later age, and most outcomes showed no significant associations with either normal thyroid function or the full range of TSH and FT4. A small association between TSH and non-verbal intelligence in girls is probably of limited clinical significance due to its small effect size. These findings suggest early developmental delays may be compensated for over time, and that perhaps only marked deviations in maternal thyroid function may have lasting impact, underscoring the need for long-term studies focused on clinically overt dysfunction and earlier identification of women at risk, ideally before conception.

## CRediT authorship contribution statement

**Sarai M. Keestra:** Writing – original draft, Methodology, Formal analysis, Conceptualization. **Susanne R. de Rooij:** Writing – review & editing, Project administration, Methodology, Funding acquisition. **Tessa J. Roseboom:** Writing – review & editing, Supervision, Conceptualization. **Marsh Königs:** Writing – review & editing, Methodology. **Tanja GM. Vrijkotte:** Writing – review & editing, Project administration, Methodology, Funding acquisition. **Martijn J.J. Finken:** Writing – review & editing, Supervision, Conceptualization.

## Funding

Financial support for the Amsterdam Born Children and their Development study was granted by the Netherlands Organization for Health Research and Development (ZonMw), The Hague, the Public Health Service and Municipal Council of Amsterdam, the Academic Medical Centre and Sarphati Amsterdam. S.K. is supported by the Amsterdam Reproduction & Development Institute Moving Forward Grant and a MD/PhD Grant from the Amsterdam UMC.

## Declaration of competing interest

The authors declare that they have no known competing financial interests or personal relationships that could have appeared to influence the work reported in this paper.

## Data Availability

The data in this article are part of the Amsterdam Born Children and their Development cohort: a multi-ethnic birth study, which follows the health, growth and development of approximately 8000 children born in Amsterdam. For more information about data access please inquire at abcd@amsterdamumc.nl.
