# Supplementary material for: Maternal thyroid function in the first half of pregnancy and neurodevelopmental outcomes in early adolescence in the Amsterdam Born Children and their Development (ABCD) cohort
Source: Compr Psychoneuroendocrinol. 2025 Dec 22;25:100333. doi: 10.1016/j.cpnec.2025.100333 (PMC12808570; doi:10.1016/j.cpnec.2025.100333)
Supplement: Multimedia component 1 [file mmc1.docx]

Supplementary 1

# Detailed visual information on variable preparation

## The division of the sample into hypo-, eu- and hyperthyroxinaemia based on the 10th and 90th percentiles of free thyroxine levels in the first 20 weeks of pregnancy

Based on our previous publications (for example (Finken et al. 2013)) we are aware that FT4 declines over time in the first trimester and that before categorisation, it is important to standardise the FT4 levels to the same date. Below a visualisation of FT4 values.


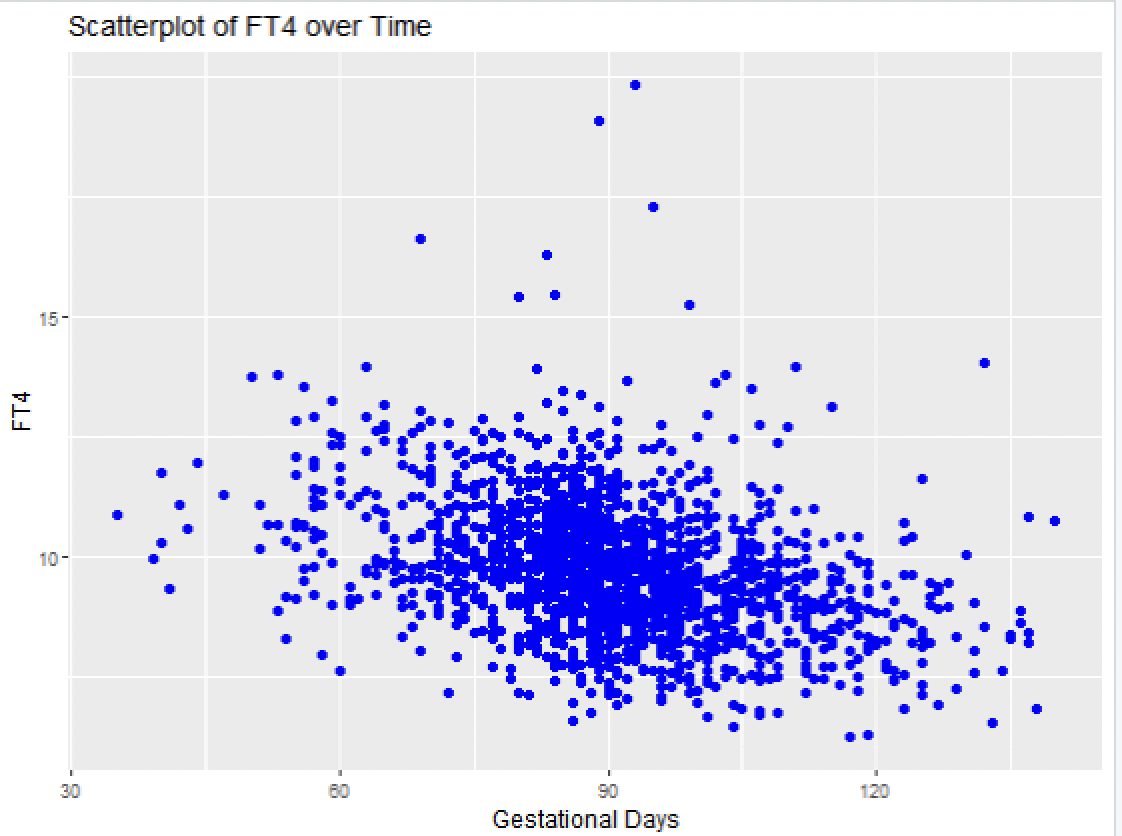


As the foetal thyroid axis becomes active itself around twenty weeks, we have excluded late testers (>20 weeks) from our analyses. We ran a linear model in a thyroid disease-free population that identified a significant linear decline in FT4 during the first 20 weeks of pregnancy of -0.033 pmol/L per gestational day and thus we standardize all participants to day 89, which is the median of our dataset. Note we did not exclude outliers at this stage as they are of interest to our analysis.


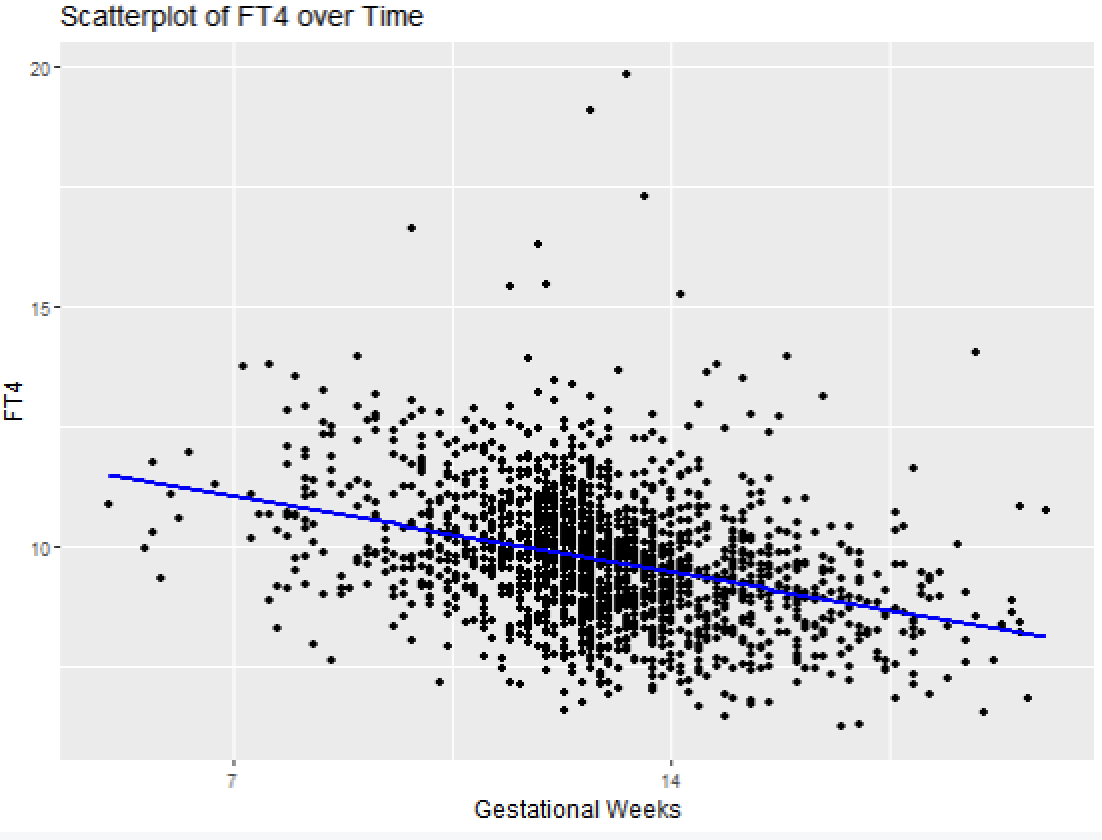


We then categorised participants in the lowest and highest 10th and 90th percentile of the FT4 distribution as cut-offs, which we then employed for further analysis.


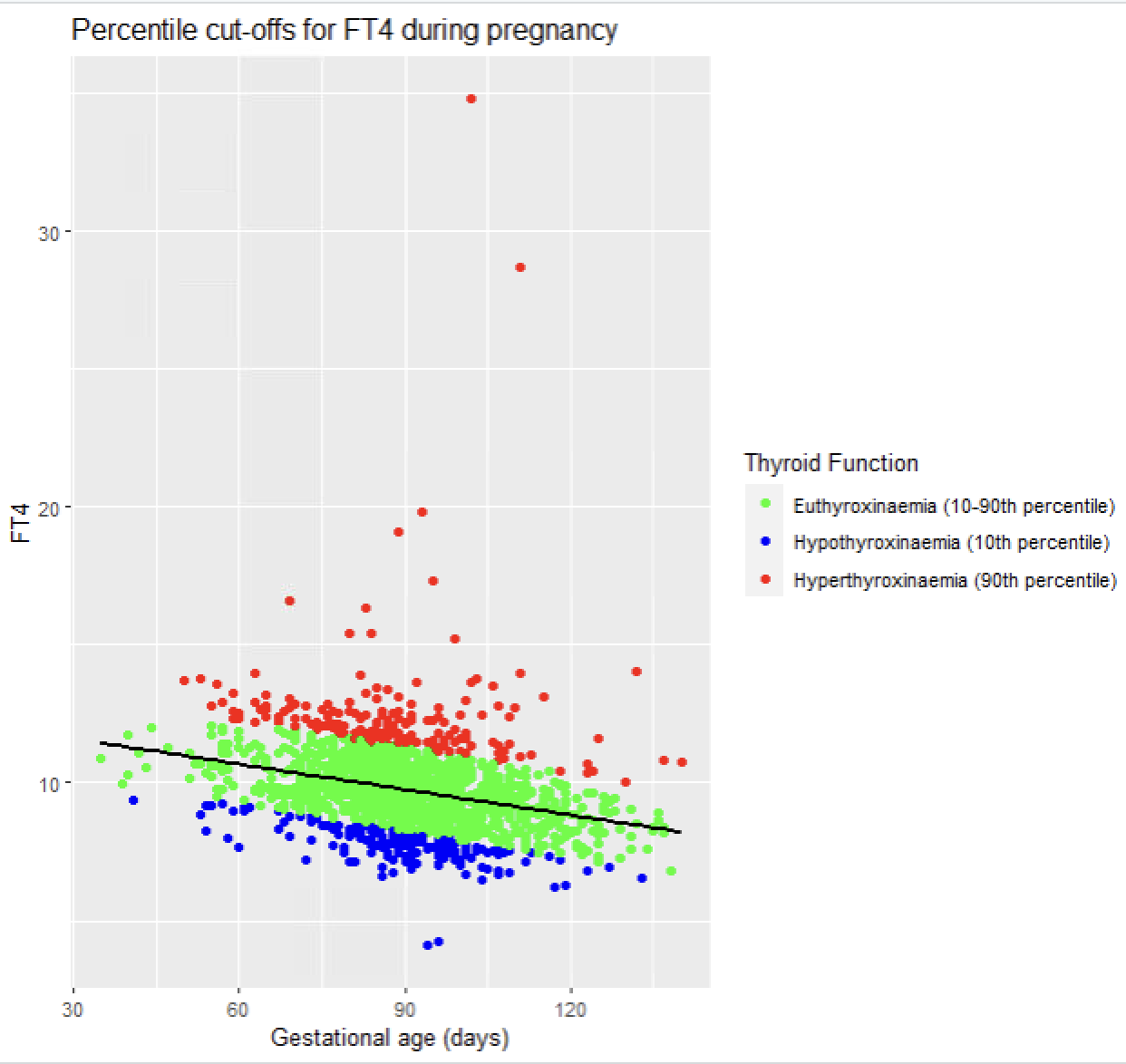


Similarly we also categorised participants based on their log-transformed thyrotropin levels, but we did not standardize for gestational age here as there was no relationship over time.


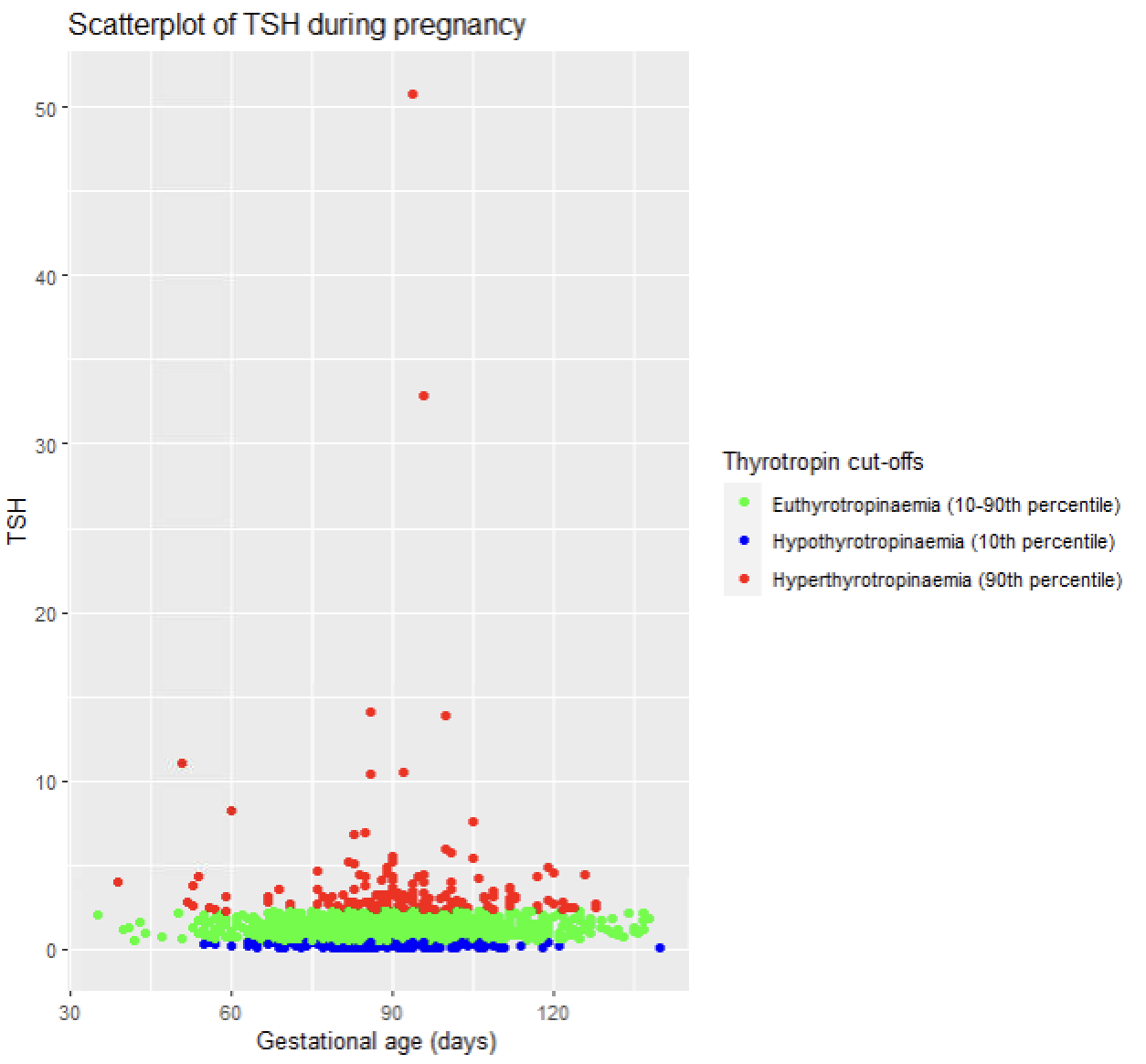


## Missing covariates before imputation using MICE package

We imputed missing covariates (<15%) using predictive mean matching through the MICE package below. Note that only few covariates we use are missing before imputation.


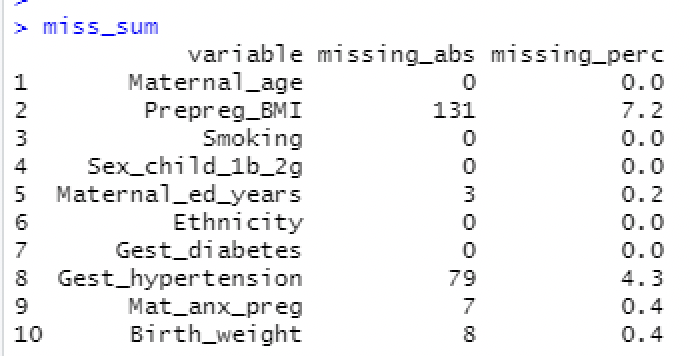


## Overview of the missing outcome variables

Overall, at least 1824 mother child-child dyads had at least one neurodevelopmental outcome available after applying exclusion criteria as described in the main text.

|  | mean | sd | min | max | n_cases | n_missing |
| --- | --- | --- | --- | --- | --- | --- |
| RAVEN_Non_Verbal_Intelligence | 15.6 | 5.86 | 2 | 28 | 1059 | 765 |
| SOPT_total_errorscore_mean | 0.2 | 0.1 | 0 | 0.73 | 655 | 1169 |
| BRIEF_Behavioural_Regulation_Index | 12.39 | 3.16 | 9 | 26 | 1501 | 323 |
| BRIEF_Metacognition_Index | 25.89 | 6.11 | 15 | 44 | 1501 | 323 |
| SURPS_Internalising_Scale | 1.6 | 0.38 | 1 | 3.93 | 1474 | 350 |
| SURPS_Risk_Taking_Behaviour_Scale | 2.32 | 0.48 | 1 | 4 | 1474 | 350 |
| SDQ_Mother_Externalising_Scale | 3.47 | 3.1 | 0 | 17 | 1509 | 315 |
| SDQ_Mother_Internalising_Scale | 2.76 | 2.84 | 0 | 17 | 1509 | 315 |
| SDQ_Teacher_Externalising_Scale | 2.43 | 3.05 | 0 | 17 | 1155 | 669 |
| SDQ_Teacher_Internalising_Scale | 1.93 | 2.36 | 0 | 13 | 1155 | 669 |
| SDQ_Self_Externalising_Scale | 4.66 | 3.05 | 0 | 16 | 1678 | 146 |
| SDQ_Self_Internalising_Scale | 2.96 | 2.52 | 0 | 14 | 1677 | 147 |

## Model choice

|  | Type of data & overdispersion | Model choice |
| --- | --- | --- |
| RAVEN_Non_Verbal_Intelligence | Count data, overdispersion | Negative binomial regression |
| SOPT_total_errorscore_mean | Proportion data | Beta regression |
| BRIEF_Behavioural_Regulation_Index | Count data, no overdispersion | Poisson regression |
| BRIEF_Metacognition_Index | Count data, overdispersion | Negative binomial regression |
| SURPS_Internalising_Scale | Continuous data | Linear regression |
| SURPS_Risk_Taking_Behaviour_Scale | Continuous data | Linear regression |
| SDQ_Mother_Externalising_Scale | Count data, overdispersion | Negative binomial regression |
| SDQ_Mother_Internalising_Scale | Count data, overdispersion | Negative binomial regression |
| SDQ_Teacher_Externalising_Scale | Count data, overdispersion | Negative binomial regression |
| SDQ_Teacher_Internalising_Scale | Count data, overdispersion | Negative binomial regression |
| SDQ_Self_Externalising_Scale | Count data, overdispersion | Negative binomial regression |
| SDQ_Self_Internalising_Scale | Count data, overdispersion | Negative binomial regression |

## Selective drop-out: comparison between the neurodevelopmental follow-up with the original ABCD cohort

| Variable | Original.Population | Selected.Population | P-value |
| --- | --- | --- | --- |
| Number of Mother-Child Dyads | 3871 | 1824 | - |
| Girls n (%) | 1941 (51.05%) | 944 (51.75%) | 0.623 |
| Maternal Age During Pregnancy mean (SD) | 31.11 (4.68) | 32.03 (4.07) | <0.01 |
| Maternal Education Years mean (SD) | 9.36 (3.74) | 10.3 (3.4) | <0.01 |
| Parity mean (SD) | 0.57 (0.81) | 0.52 (0.75) | 0.023 |
| Smoking During Pregnancy n (%) | 356 (9.21%) | 121 (6.63%) | <0.01 |
| Dutch Ethnicity n (%) | 2332 (60.24%) | 1334 (73.14%) | <0.01 |
| Pre-Pregnancy BMI mean (SD) | 22.83 (3.92) | 22.64 (3.51) | 0.076 |
| Birth Weight Child mean (SD) | 3436.14 (628.62) | 3507.17 (544.53) | <0.01 |
| Gestational Age at Birth mean (SD) | 39.17 (3.05) | 39.56 (1.66) | <0.01 |
| FT4 mean (SD) | 9.69 (1.7) | 9.74 (1.56) | 0.265 |
| FastTSH mean (SD) | 1.4 (2.63) | 1.39 (1.71) | 0.747 |
| Thyroid Testing Day mean (SD) | 90.85 (16.1) | 90.46 (14.77) | 0.36 |

We used a chi-squared test to compare categorical variables and a t-test to compare continuous variables
