# Supplementary material for: Maternal thyroid function in the first half of pregnancy and neurodevelopmental outcomes in early adolescence in the Amsterdam Born Children and their Development (ABCD) cohort
Source: Compr Psychoneuroendocrinol. 2025 Dec 22;25:100333. doi: 10.1016/j.cpnec.2025.100333 (PMC12808570; doi:10.1016/j.cpnec.2025.100333)
Supplement: Multimedia component 2 [file mmc2.docx]

Supplementary 2

## Sensitivity analysis of all mother-child dyads, including mothers that have overt hypo- or hyperthyroidism, taking thyroid hormone replacement therapy at the first antenatal visit, or who had children that were born preterm (<32 weeks) or at very low birth weight (<1500g).

| Neurodevelopmental outcome | Unadjusted models | | | | Adjusted models | | | | | |
| --- | --- | --- | --- | --- | --- | --- | --- | --- | --- | --- |
|  | Thyroid Parameter^1^ | estimate | standard error | p-value | estimate | standard error | p-value | p-value after FDR correction^2^ | Confidence interval lower bound | Confidence interval higher bound |
| Non-verbal intelligence | FT4 | -0.02 | 0.01 | 0.22 | -0.02 | 0.01 | 0.11 | 0.23 | -0.04 | 0 |
| Non-verbal intelligence | **TSH** | 0.04 | 0.01 | 0 | 0.03 | 0.01 | 0.01 | 0.04 | 0.01 | 0.05 |
| Executive working memory | FT4 | -0.02 | 0.03 | 0.54 | 0 | 0.03 | 0.9 | 0.9 | -0.06 | 0.06 |
| Executive working memory | TSH | -0.02 | 0.03 | 0.35 | -0.02 | 0.03 | 0.5 | 0.66 | -0.08 | 0.04 |
| Behavioural regulation | FT4 | 0.01 | 0.01 | 0.49 | 0.01 | 0.01 | 0.47 | 0.88 | -0.01 | 0.03 |
| Behavioural regulation | TSH | 0 | 0.01 | 0.8 | 0 | 0.01 | 0.62 | 0.88 | -0.02 | 0.02 |
| Metacognition | FT4 | 0 | 0.01 | 0.85 | 0 | 0.01 | 0.88 | 0.88 | -0.02 | 0.02 |
| Metacognition | TSH | 0 | 0.01 | 0.49 | 0 | 0.01 | 0.69 | 0.88 | -0.02 | 0.02 |
| Internalising traits | FT4 | 0 | 0.01 | 0.84 | 0 | 0.01 | 0.85 | 0.88 | -0.02 | 0.02 |
| Internalising traits | TSH | -0.02 | 0.01 | 0.03 | -0.02 | 0.01 | 0.1 | 0.78 | -0.04 | 0 |
| Risk taking behaviour | FT4 | 0.02 | 0.01 | 0.14 | 0.01 | 0.01 | 0.7 | 0.88 | -0.01 | 0.03 |
| Risk taking behaviour | TSH | -0.01 | 0.01 | 0.58 | -0.01 | 0.01 | 0.61 | 0.88 | -0.03 | 0.01 |
| Mother-Reported Externalizing Problems | FT4 | -0.02 | 0.03 | 0.45 | -0.02 | 0.03 | 0.55 | 0.66 | -0.08 | 0.04 |
| Mother-Reported Externalizing Problems | TSH | 0.02 | 0.03 | 0.52 | 0.02 | 0.03 | 0.38 | 0.64 | -0.04 | 0.08 |
| Mother-Reported Internalizing Problems | FT4 | -0.04 | 0.03 | 0.15 | -0.03 | 0.03 | 0.34 | 0.64 | -0.09 | 0.03 |
| Mother-Reported Internalizing Problems | TSH | 0.02 | 0.03 | 0.56 | 0.03 | 0.03 | 0.26 | 0.64 | -0.03 | 0.09 |
| Teacher-Reported Externalizing Problems | FT4 | -0.07 | 0.04 | 0.14 | -0.05 | 0.04 | 0.23 | 0.64 | -0.13 | 0.03 |
| Teacher-Reported Externalizing Problems | TSH | -0.03 | 0.04 | 0.49 | -0.01 | 0.04 | 0.73 | 0.8 | -0.09 | 0.07 |
| Teacher-Reported Internalizing Problems | FT4 | -0.12 | 0.04 | 0 | -0.09 | 0.04 | 0.03 | 0.31 | -0.17 | -0.01 |
| Teacher-Reported Internalizing Problems | TSH | 0.03 | 0.04 | 0.45 | 0.03 | 0.04 | 0.48 | 0.64 | -0.05 | 0.11 |
| Self-Reported Externalizing Problems | FT4 | 0.01 | 0.02 | 0.75 | 0 | 0.02 | 0.92 | 0.92 | -0.04 | 0.04 |
| Self-Reported Externalizing Problems | TSH | -0.01 | 0.02 | 0.47 | -0.01 | 0.02 | 0.43 | 0.64 | -0.05 | 0.03 |
| Self-Reported Internalizing Problems | FT4 | -0.04 | 0.02 | 0.05 | -0.05 | 0.02 | 0.05 | 0.31 | -0.09 | -0.01 |
| Self-Reported Internalizing Problems | TSH | 0.01 | 0.02 | 0.69 | 0.02 | 0.02 | 0.3 | 0.64 | -0.02 | 0.06 |

1: FT4 was standardized for the median gestational day of testing (89 days), TSH was log-transformed and both variables were scaled before analysis;
