# Supplementary material for: Maternal thyroid function in the first half of pregnancy and neurodevelopmental outcomes in early adolescence in the Amsterdam Born Children and their Development (ABCD) cohort
Source: Compr Psychoneuroendocrinol. 2025 Dec 22;25:100333. doi: 10.1016/j.cpnec.2025.100333 (PMC12808570; doi:10.1016/j.cpnec.2025.100333)
Supplement: Multimedia component 3 [file mmc3.docx]

**Supplementary 3**

# Analyses using the 10th and 90th percentiles cut-offs for FT4 and TSH respectively

## Overview of characteristics of the different thyroid cut-off groups

| Variable | Entire sample | Hypo-thyroxinaemia (lowest 10th percentile FT4) | Eu-thyroxinaemia | Hyper-thyroxinaemia  (highest 90th percentile FT4) | Hypo-thyrotropinaemia  (lowest 10th percentile log TSH) | Eu-thyrotropinaemia | Hyper-thyrotropinaemia  (highest 90th percentile log TSH) |
| --- | --- | --- | --- | --- | --- | --- | --- |
| Number of Mother-Child Dyads | 1824 | 183 | 1458 | 183 | 180 | 1462 | 182 |
| Girls n (%) | 944 (51.75%) | 97 (53.01%) | 760 (52.13%) | 87 (47.54%) | 99 (55%) | 753 (51.5%) | 92 (50.55%) |
| Age of Child in Years At Testing mean (SD) | 11.56 (0.31) | 11.61 (0.33) | 11.56 (0.31) | 11.54 (0.3) | 11.54 (0.29) | 11.56 (0.31) | 11.59 (0.33) |
| Maternal Age During Pregnancy mean (SD) | 32.03 (4.07) | 32.08 (4.15) | 32.02 (4.11) | 32.09 (3.72) | 32.3 (4.34) | 32.05 (4.06) | 31.67 (3.95) |
| Parity mean (SD) | 0.52 (0.75) | 0.62 (0.88) | 0.51 (0.74) | 0.55 (0.7) | 0.74 (0.9) | 0.51 (0.73) | 0.44 (0.68) |
| Dutch ethnicity n (%) | 1334 (73.14%) | 137 (74.86%) | 1071 (73.46%) | 126 (68.85%) | 110 (61.11%) | 1086 (74.28%) | 138 (75.82%) |
| Maternal Education Years mean (SD) | 10.29 (3.4) | 9.56 (3.53) | 10.37 (3.37) | 10.39 (3.45) | 9.82 (3.9) | 10.32 (3.35) | 10.54 (3.21) |
| Smoking During Pregnancy n (%) | 121 (6.63%) | 23 (12.57%) | 89 (6.1%) | 9 (4.92%) | 15 (8.33%) | 97 (6.63%) | 9 (4.95%) |
| Pre-Pregnancy BMI mean (SD) | 22.67 (3.42) | 23.85 (4.17) | 22.57 (3.31) | 22.26 (3.26) | 23.18 (3.69) | 22.62 (3.4) | 22.58 (3.29) |
| Birth Weight Child mean (SD) | 3507.52 (543.52) | 3534.33 (639.17) | 3507.05 (531.02) | 3484.4 (540.17) | 3521.69 (555.48) | 3501.95 (544.39) | 3538.23 (526) |
| Thyroid Testing Gestational Week mean (SD) | 12.93 (2.12) | 12.67 (2.01) | 13 (2.1) | 12.6 (2.36) | 12.56 (1.96) | 12.95 (2.12) | 13.14 (2.26) |
| FT4 mean (SD) | 9.74 (1.56) | 7.78 (0.71) | 9.64 (0.9) | 12.51 (2.42) | 11.03 (2.88) | 9.68 (1.23) | 8.96 (1.34) |
| FastTSH mean (SD) | 1.39 (1.71) | 2.5 (4.62) | 1.3 (0.85) | 0.93 (0.73) | 0.24 (0.13) | 1.23 (0.47) | 3.8 (4.5) |
| Anti-TPO Positive n (%) | 114 (6.25%) | 37 (20.22%) | 66 (4.53%) | 11 (6.01%) | 7 (3.89%) | 64 (4.38%) | 43 (23.63%) |

## Hypothyroxinaemia based on being in the lowest 10th percentile for FT4 (<8.28 pmol/L)

| Neurodevelopmental outcome | Estimate | Standard Error | P-Value | Lower CI | Upper CI | FDR-Corrected P-Value |
| --- | --- | --- | --- | --- | --- | --- |
| Non-verbal intelligence | 0.03 | 0.04 | 0.45 | -0.05 | 0.11 | 0.76 |
| Executive working memory | -0.02 | 0.08 | 0.76 | -0.19 | 0.14 | 0.76 |
| Behavioural Regulation | -0.01 | 0.03 | 0.78 | -0.06 | 0.04 | 0.87 |
| Metacognition | 0 | 0.02 | 0.87 | -0.04 | 0.04 | 0.87 |
| Internalising behaviour | -0.03 | 0.03 | 0.37 | -0.1 | 0.04 | 0.74 |
| Risk Taking Behaviour | -0.05 | 0.04 | 0.23 | -0.14 | 0.03 | 0.74 |
| Mother-Reported Externalising Problems | 0.06 | 0.08 | 0.48 | -0.1 | 0.22 | 0.77 |
| Mother-Reported Internalising Problems | 0.08 | 0.09 | 0.36 | -0.09 | 0.25 | 0.77 |
| Teacher-Reported Externalising Problems | 0.08 | 0.13 | 0.56 | -0.18 | 0.33 | 0.77 |
| Teacher-Reported Internalising Problems | 0.04 | 0.12 | 0.77 | -0.2 | 0.27 | 0.77 |
| Self-Reported Externalising Problems | -0.04 | 0.06 | 0.5 | -0.15 | 0.07 | 0.77 |
| Self-Reported Internalising Problems | -0.02 | 0.07 | 0.77 | -0.16 | 0.12 | 0.77 |

## Hyperthyroxinaemia based on being in the highest 90th percentile for FT4 (>11.33 pmol/L)

| Neurodevelopmental outcome | Estimate | Standard Error | P-Value | Lower CI | Upper CI | FDR-Corrected P-Value |
| --- | --- | --- | --- | --- | --- | --- |
| Non-verbal intelligence | -0.08 | 0.04 | 0.05 | -0.16 | 0.00 | 0.10 |
| Executive working memory | -0.04 | 0.08 | 0.65 | -0.20 | 0.13 | 0.65 |
| Behavioural Regulation | 0.02 | 0.02 | 0.51 | -0.03 | 0.06 | 0.87 |
| Metacognition | 0.00 | 0.02 | 0.82 | -0.03 | 0.04 | 0.87 |
| Internalising behaviour | -0.03 | 0.03 | 0.32 | -0.10 | 0.03 | 0.87 |
| Risk Taking Behaviour | -0.01 | 0.04 | 0.87 | -0.09 | 0.08 | 0.87 |
| Mother-Reported Externalising Problems | 0.01 | 0.08 | 0.94 | -0.15 | 0.16 | 0.94 |
| Mother-Reported Internalising Problems | -0.15 | 0.09 | 0.09 | -0.32 | 0.03 | 0.18 |
| Teacher-Reported Externalising Problems | 0.05 | 0.13 | 0.72 | -0.21 | 0.31 | 0.94 |
| Teacher-Reported Internalising Problems | **-0.27** | **0.13** | **0.04** | **-0.52** | **-0.01** | **0.12** |
| Self-Reported Externalising Problems | -0.01 | 0.06 | 0.90 | -0.12 | 0.10 | 0.94 |
| Self-Reported Internalising Problems | **-0.19** | **0.07** | **0.01** | **-0.33** | **-0.04** | **0.06** |

## Hypothyrotropinaemia based on being in the lowest 10th percentile for TSH (<0.45 mU/l)

| Neurodevelopmental outcome | Estimate | Standard Error | P-Value | Lower CI | Upper CI | FDR-Corrected P-Value |
| --- | --- | --- | --- | --- | --- | --- |
| Non-verbal intelligence | -0.04 | 0.04 | 0.35 | -0.12 | 0.04 | 0.48 |
| Executive working memory | 0.06 | 0.08 | 0.48 | -0.11 | 0.23 | 0.48 |
| Behavioural Regulation | -0.01 | 0.03 | 0.83 | -0.06 | 0.04 | 0.83 |
| Metacognition | 0.02 | 0.02 | 0.40 | -0.02 | 0.06 | 0.80 |
| Internalising behaviour | 0.02 | 0.03 | 0.60 | -0.05 | 0.09 | 0.80 |
| Risk Taking Behaviour | 0.03 | 0.04 | 0.52 | -0.06 | 0.11 | 0.80 |
| Mother-Reported Externalising Problems | 0.02 | 0.08 | 0.83 | -0.15 | 0.18 | 0.83 |
| Mother-Reported Internalising Problems | -0.05 | 0.09 | 0.61 | -0.23 | 0.13 | 0.83 |
| Teacher-Reported Externalising Problems | 0.08 | 0.14 | 0.54 | -0.19 | 0.36 | 0.83 |
| Teacher-Reported Internalising Problems | -0.04 | 0.13 | 0.78 | -0.30 | 0.22 | 0.83 |
| Self-Reported Externalising Problems | 0.08 | 0.06 | 0.15 | -0.03 | 0.19 | 0.75 |
| Self-Reported Internalising Problems | -0.08 | 0.07 | 0.25 | -0.23 | 0.06 | 0.75 |

## Hyperthyrotropinaemia based on being in the highest 90th percentile for TSH (>2.28 mU/l)

| Neurodevelopmental outcome | Estimate | Standard Error | P-Value | Lower CI | Upper CI | FDR-Corrected P-Value |
| --- | --- | --- | --- | --- | --- | --- |
| Non-verbal intelligence | 0.05 | 0.04 | 0.24 | -0.03 | 0.13 | 0.48 |
| Executive working memory | 0.01 | 0.08 | 0.86 | -0.14 | 0.17 | 0.86 |
| Behavioural Regulation | -0.02 | 0.03 | 0.51 | -0.07 | 0.03 | 0.51 |
| Metacognition | 0.01 | 0.02 | 0.48 | -0.03 | 0.05 | 0.51 |
| Internalising behaviour | -0.06 | 0.03 | 0.09 | -0.12 | 0.01 | 0.36 |
| Risk Taking Behaviour | -0.04 | 0.04 | 0.31 | -0.13 | 0.04 | 0.51 |
| Mother-Reported Externalising Problems | -0.01 | 0.08 | 0.93 | -0.17 | 0.15 | 0.93 |
| Mother-Reported Internalising Problems | 0.03 | 0.09 | 0.72 | -0.14 | 0.21 | 0.93 |
| Teacher-Reported Externalising Problems | -0.08 | 0.13 | 0.54 | -0.34 | 0.18 | 0.93 |
| Teacher-Reported Internalising Problems | -0.11 | 0.12 | 0.38 | -0.35 | 0.13 | 0.93 |
| Self-Reported Externalising Problems | -0.05 | 0.06 | 0.42 | -0.16 | 0.07 | 0.93 |
| Self-Reported Internalising Problems | 0.02 | 0.07 | 0.8 | -0.12 | 0.16 | 0.93 |
