# Supplementary material for: Maternal thyroid function in the first half of pregnancy and neurodevelopmental outcomes in early adolescence in the Amsterdam Born Children and their Development (ABCD) cohort
Source: Compr Psychoneuroendocrinol. 2025 Dec 22;25:100333. doi: 10.1016/j.cpnec.2025.100333 (PMC12808570; doi:10.1016/j.cpnec.2025.100333)
Supplement: Multimedia component 4 [file mmc4.docx]

Supplementary 4

## Interaction term for sex and the respective thyroid parameter

| Neurodevelopmental outcome | Thyroid Parameter^1^ | estimate | standard error | p-value | Confidence interval lower bound | Confidence interval higher bound |
| --- | --- | --- | --- | --- | --- | --- |
| Non-verbal intelligence | **TSH** | **0.05** | **0.03** | **0.05** | **-0.01** | **0.11** |
| Non-verbal intelligence | FT4 | -0.02 | 0.03 | 0.34 | -0.08 | 0.04 |
| Executive working memory | TSH | -0.06 | 0.05 | 0.26 | -0.16 | 0.04 |
| Executive working memory | FT4 | 0.04 | 0.05 | 0.49 | -0.06 | 0.14 |
| Behavioural regulation | TSH | 0.02 | 0.02 | 0.11 | -0.02 | 0.06 |
| Behavioural regulation | FT4 | -0.02 | 0.02 | 0.24 | -0.06 | 0.02 |
| Metacognition | TSH | 0.01 | 0.01 | 0.65 | -0.01 | 0.03 |
| Metacognition | FT4 | 0 | 0.01 | 0.72 | -0.02 | 0.02 |
| Internalising traits | TSH | 0.03 | 0.02 | 0.15 | -0.01 | 0.07 |
| Internalising traits | FT4 | 0.01 | 0.02 | 0.75 | -0.03 | 0.05 |
| Risk taking behaviour | TSH | 0 | 0.03 | 0.89 | -0.06 | 0.06 |
| Risk taking behaviour | FT4 | 0.01 | 0.03 | 0.69 | -0.05 | 0.07 |
| Mother-Reported Externalizing Problems | TSH | 0.10 | 0.05 | 0.05 | 0 | 0.2 |
| Mother-Reported Externalizing Problems | **FT4** | **-0.11** | **0.05** | **0.03** | **-0.21** | **-0.01** |
| Mother-Reported Internalizing Problems | TSH | 0.03 | 0.06 | 0.55 | -0.09 | 0.15 |
| Mother-Reported Internalizing Problems | FT4 | -0.03 | 0.06 | 0.6 | -0.15 | 0.09 |
| Teacher-Reported Externalizing Problems | TSH | -0.07 | 0.09 | 0.45 | -0.25 | 0.11 |
| Teacher-Reported Externalizing Problems | FT4 | -0.05 | 0.09 | 0.53 | -0.23 | 0.13 |
| Teacher-Reported Internalizing Problems | TSH | 0.03 | 0.08 | 0.75 | -0.13 | 0.19 |
| Teacher-Reported Internalizing Problems | FT4 | -0.06 | 0.08 | 0.49 | -0.22 | 0.1 |
| Self-Reported Externalizing Problems | **TSH** | **0.06** | **0.03** | **0.07** | **0.00** | **0.12** |
| Self-Reported Externalizing Problems | FT4 | 0.01 | 0.04 | 0.78 | -0.07 | 0.09 |
| Self-Reported Internalizing Problems | **TSH** | **0.09** | **0.04** | **0.04** | **0.01** | **0.17** |
| Self-Reported Internalizing Problems | FT4 | -0.02 | 0.05 | 0.72 | -0.12 | 0.08 |

1: FT4 was standardized for the median gestational day of testing (89 days); TSH was log-transformed; Both FT4 and TSH were scaled before analysis
