# Supplementary material for: Maternal thyroid function in the first half of pregnancy and neurodevelopmental outcomes in early adolescence in the Amsterdam Born Children and their Development (ABCD) cohort
Source: Compr Psychoneuroendocrinol. 2025 Dec 22;25:100333. doi: 10.1016/j.cpnec.2025.100333 (PMC12808570; doi:10.1016/j.cpnec.2025.100333)
Supplement: Multimedia component 5 [file mmc5.docx]

Supplementary 5

## Sex-stratified analysis for boys only, FDR adjustment after all models are run

| Neurodevelopmental outcome | Unadjusted models | | | | Adjusted models | | | | | |
| --- | --- | --- | --- | --- | --- | --- | --- | --- | --- | --- |
|  | Thyroid Parameter^1^ | estimate | standard error | p-value | estimate | standard error | p-value | p-value after FDR correction^2^ | Confidence interval lower bound | Confidence interval higher bound |
| Non-verbal intelligence | FT4 | -0.02 | 0.02 | 0.32 | -0.02 | 0.02 | 0.20 | 0.82 | -0.06 | 0.02 |
| Non-verbal intelligence | TSH | 0.02 | 0.02 | 0.24 | 0.01 | 0.02 | 0.57 | 0.98 | -0.03 | 0.05 |
| Executive working memory | FT4 | -0.04 | 0.04 | 0.33 | 0.00 | 0.04 | 0.98 | 0.98 | -0.08 | 0.08 |
| Executive working memory | TSH | 0.00 | 0.04 | 0.92 | 0.01 | 0.04 | 0.87 | 0.98 | -0.07 | 0.09 |
| Behavioural regulation | FT4 | 0.01 | 0.01 | 0.40 | 0.01 | 0.01 | 0.40 | 0.88 | -0.01 | 0.03 |
| Behavioural regulation | TSH | -0.01 | 0.01 | 0.54 | -0.01 | 0.01 | 0.61 | 0.88 | -0.03 | 0.01 |
| Metacognition | FT4 | 0.00 | 0.01 | 0.75 | 0.00 | 0.01 | 0.57 | 0.88 | -0.02 | 0.02 |
| Metacognition | TSH | 0.00 | 0.01 | 0.72 | 0.00 | 0.01 | 0.83 | 0.88 | -0.02 | 0.02 |
| Internalising traits | **FT4** | **-0.03** | **0.01** | **0.04** | **-0.03** | **0.01** | **0.05** | **0.20** | **-0.05** | **-0.01** |
| Internalising traits | **TSH** | **-0.02** | **0.01** | **0.08** | **-0.03** | **0.01** | **0.04** | **0.20** | **-0.05** | **-0.01** |
| Risk taking behaviour | FT4 | 0.00 | 0.02 | 0.99 | 0.00 | 0.02 | 0.88 | 0.88 | -0.04 | 0.04 |
| Risk taking behaviour | TSH | 0.00 | 0.02 | 0.84 | 0.00 | 0.02 | 0.88 | 0.88 | -0.04 | 0.04 |
| Mother-Reported Externalizing Problems | FT4 | 0.00 | 0.03 | 0.93 | 0.02 | 0.03 | 0.57 | 0.76 | -0.04 | 0.08 |
| Mother-Reported Externalizing Problems | TSH | -0.04 | 0.03 | 0.26 | -0.03 | 0.03 | 0.33 | 0.61 | -0.09 | 0.03 |
| Mother-Reported Internalizing Problems | FT4 | -0.04 | 0.04 | 0.28 | -0.04 | 0.04 | 0.35 | 0.61 | -0.12 | 0.04 |
| Mother-Reported Internalizing Problems | TSH | 0.00 | 0.04 | 1.00 | 0.01 | 0.04 | 0.80 | 0.80 | -0.07 | 0.09 |
| Teacher-Reported Externalizing Problems | FT4 | -0.02 | 0.05 | 0.61 | 0.03 | 0.05 | 0.50 | 0.75 | -0.07 | 0.13 |
| Teacher-Reported Externalizing Problems | TSH | -0.09 | 0.06 | 0.12 | -0.02 | 0.06 | 0.69 | 0.80 | -0.14 | 0.10 |
| Teacher-Reported Internalizing Problems | FT4 | -0.09 | 0.05 | 0.09 | -0.06 | 0.05 | 0.25 | 0.61 | -0.16 | 0.04 |
| Teacher-Reported Internalizing Problems | TSH | -0.06 | 0.06 | 0.30 | -0.06 | 0.06 | 0.31 | 0.61 | -0.18 | 0.06 |
| Self-Reported Externalizing Problems | FT4 | -0.01 | 0.02 | 0.57 | -0.01 | 0.02 | 0.77 | 0.80 | -0.05 | 0.03 |
| Self-Reported Externalizing Problems | **TSH** | **-0.04** | **0.02** | **0.10** | **-0.05** | **0.03** | **0.04** | **0.50** | **-0.11** | **0.01** |
| Self-Reported Internalizing Problems | FT4 | -0.07 | 0.03 | 0.03 | -0.05 | 0.03 | 0.15 | 0.61 | -0.11 | 0.01 |
| Self-Reported Internalizing Problems | TSH | -0.04 | 0.03 | 0.22 | -0.05 | 0.03 | 0.17 | 0.61 | -0.11 | 0.01 |

1: FT4 was standardized for the median gestational day of testing (89 days); TSH was log-transformed; both FT4 and TSH were scaled before analysis; 2 FDR correction on a domain level after running all models.
