# Supplementary material for: Maternal thyroid function in the first half of pregnancy and neurodevelopmental outcomes in early adolescence in the Amsterdam Born Children and their Development (ABCD) cohort
Source: Compr Psychoneuroendocrinol. 2025 Dec 22;25:100333. doi: 10.1016/j.cpnec.2025.100333 (PMC12808570; doi:10.1016/j.cpnec.2025.100333)
Supplement: Multimedia component 6 [file mmc6.docx]

Supplementary 6

## Sex-stratified analysis for girls only, FDR adjustment after all models are run

| Neurodevelopmental outcome | Unadjusted models | | | | Adjusted models | | | | | |
| --- | --- | --- | --- | --- | --- | --- | --- | --- | --- | --- |
|  | Thyroid Parameter^1^ | estimate | standard error | p-value | estimate | standard error | p-value | p-value after FDR correction^2^ | Confidence interval lower bound | Confidence interval higher bound |
| Non-verbal intelligence | FT4 | -0.02 | 0.02 | 0.17 | -0.03 | 0.02 | 0.11 | 0.21 | -0.07 | 0.01 |
| Non-verbal intelligence | **TSH** | **0.05** | **0.02** | **0.00** | **0.05** | **0.02** | **<0.01** | **0.02** | **0.01** | **0.09** |
| Executive working memory | FT4 | -0.02 | 0.04 | 0.62 | -0.02 | 0.04 | 0.67 | 0.67 | -0.10 | 0.06 |
| Executive working memory | TSH | -0.03 | 0.03 | 0.41 | -0.02 | 0.03 | 0.56 | 0.67 | -0.08 | 0.04 |
| Behavioural regulation | FT4 | 0.00 | 0.01 | 0.77 | 0.00 | 0.01 | 0.96 | 0.96 | -0.02 | 0.02 |
| Behavioural regulation | TSH | 0.01 | 0.01 | 0.39 | 0.01 | 0.01 | 0.22 | 0.96 | -0.01 | 0.03 |
| Metacognition | FT4 | 0.00 | 0.01 | 0.72 | 0.00 | 0.01 | 0.82 | 0.96 | -0.02 | 0.02 |
| Metacognition | TSH | 0.01 | 0.01 | 0.50 | 0.00 | 0.01 | 0.62 | 0.96 | -0.02 | 0.02 |
| Internalising traits | FT4 | 0.00 | 0.01 | 0.79 | 0.01 | 0.02 | 0.71 | 0.96 | -0.03 | 0.05 |
| Internalising traits | TSH | -0.01 | 0.01 | 0.41 | 0.00 | 0.01 | 0.74 | 0.96 | -0.02 | 0.02 |
| Risk taking behaviour | FT4 | 0.00 | 0.02 | 0.95 | 0.00 | 0.02 | 0.79 | 0.96 | -0.04 | 0.04 |
| Risk taking behaviour | TSH | 0.00 | 0.02 | 0.93 | 0.00 | 0.02 | 0.86 | 0.96 | -0.04 | 0.04 |
| Mother-Reported Externalizing Problems | FT4 | -0.08 | 0.04 | 0.04 | -0.05 | 0.04 | 0.16 | 0.38 | -0.13 | 0.03 |
| Mother-Reported Externalizing Problems | TSH | 0.05 | 0.04 | 0.21 | 0.05 | 0.04 | 0.20 | 0.40 | -0.03 | 0.13 |
| Mother-Reported Internalizing Problems | FT4 | -0.07 | 0.04 | 0.08 | -0.04 | 0.04 | 0.31 | 0.53 | -0.12 | 0.04 |
| Mother-Reported Internalizing Problems | TSH | 0.02 | 0.04 | 0.56 | 0.03 | 0.04 | 0.40 | 0.53 | -0.05 | 0.11 |
| Teacher-Reported Externalizing Problems | FT4 | -0.15 | 0.07 | 0.03 | -0.14 | 0.07 | 0.06 | 0.20 | -0.28 | 0.00 |
| Teacher-Reported Externalizing Problems | TSH | -0.01 | 0.07 | 0.87 | -0.01 | 0.07 | 0.86 | 0.86 | -0.15 | 0.13 |
| Teacher-Reported Internalizing Problems | FT4 | -0.13 | 0.06 | 0.02 | -0.10 | 0.06 | 0.07 | 0.20 | -0.22 | 0.02 |
| Teacher-Reported Internalizing Problems | TSH | 0.01 | 0.05 | 0.83 | 0.04 | 0.05 | 0.45 | 0.54 | -0.06 | 0.14 |
| Self-Reported Externalizing Problems | FT4 | -0.02 | 0.03 | 0.48 | -0.01 | 0.03 | 0.83 | 0.86 | -0.07 | 0.05 |
| Self-Reported Externalizing Problems | TSH | 0.02 | 0.02 | 0.39 | 0.02 | 0.02 | 0.37 | 0.53 | -0.02 | 0.06 |
| Self-Reported Internalizing Problems | **FT4** | **-0.08** | **0.03** | **0.01** | **-0.07** | **0.03** | **0.03** | **0.20** | **-0.13** | **-0.01** |
| Self-Reported Internalizing Problems | **TSH** | **0.04** | **0.03** | **0.11** | **0.06** | **0.03** | **0.04** | **0.20** | **0.00** | **0.12** |

1: FT4 was standardized for the median gestational day of testing (89 days); TSH was log-transformed; both FT4 and TSH were scaled before analysis; 2 FDR correction on a domain level after running all models.
