# Supplementary material for: Maternal thyroid function in the first half of pregnancy and neurodevelopmental outcomes in early adolescence in the Amsterdam Born Children and their Development (ABCD) cohort
Source: Compr Psychoneuroendocrinol. 2025 Dec 22;25:100333. doi: 10.1016/j.cpnec.2025.100333 (PMC12808570; doi:10.1016/j.cpnec.2025.100333)
Supplement: Multimedia component 7 [file mmc7.docx]

Supplementary 7

## Extensive results of the neurodevelopmental outcomes of children born from mothers with clinical thyroid dysfunction in the first 20 weeks of pregnancy. Extensive results of the neurodevelopmental outcomes of children born from mothers with clinical thyroid dysfunction in the first 20 weeks of pregnancy.

| Variable | Euthyroid | Overt Hypothyroidism | Overt Hyperthyroidism | Subclinical Hypothyroidism | Subclinical Hyperthyroidism | Clinical Hypothyroxinaemia | Clinical Hyperthyroxinaemia |
| --- | --- | --- | --- | --- | --- | --- | --- |
| Number of Mother-Child Dyads | 1648 | 13 | 12 | 56 | 31 | 36 | 28 |
| Girls | 851 (51.64%) | 6 (46.15%) | 8 (66.67%) | 30 (53.57%) | 22 (70.97%) | 18 (50%) | 9 (32.14%) |
| Age of Child (Years) | 11.56 (0.31) | 11.65 (0.25) | 11.51 (0.31) | 11.63 (0.34) | 11.6 (0.27) | 11.65 (0.32) | 11.48 (0.2) |
| Maternal Age During Pregnancy (Years) | 32 (4.07) | 33 (1.73) | 32.67 (2.39) | 31.66 (3.72) | 32.52 (4.86) | 32.67 (4.87) | 32.43 (4.38) |
| Parity | 0.51 (0.73) | 0.38 (0.65) | 0.75 (0.97) | 0.43 (0.71) | 0.81 (0.95) | 0.89 (1.12) | 0.79 (0.69) |
| Dutch ethnicity | 1210 (73.42%) | 12 (92.31%) | 8 (66.67%) | 42 (75%) | 14 (45.16%) | 25 (69.44%) | 23 (82.14%) |
| Maternal Education (Years) | 10.33 (3.37) | 9.62 (3.33) | 10.75 (2.93) | 10.79 (2.75) | 9.03 (5.19) | 8.67 (3.83) | 10.75 (2.98) |
| Smoking During Pregnancy | 103 (6.25%) | 1 (7.69%) | 1 (8.33%) | 4 (7.14%) | 0 (0%) | 10 (27.78%) | 2 (7.14%) |
| Pre-Pregnancy BMI (kg/m^2^) | 22.64 (3.4) | 21.77 (2.09) | 22.57 (2.52) | 22.13 (2.52) | 22.31 (2.51) | 25.54 (5.14) | 22.52 (4) |
| Birth Weight Child (g) | 3512.45 (535.52) | 3616.15 (427.58) | 3392.08 (614.45) | 3508.39 (565.88) | 3439.35 (684.77) | 3361.32 (776.56) | 3477.96 (462.71) |
| Thyroid Testing Gestational Week | 12.92 (2.09) | 13.23 (1.83) | 13.17 (1.95) | 12.62 (2.34) | 13.26 (2.35) | 13.53 (2.4) | 13 (2.94) |
| FT4 (pmol/L) | 9.71 (1.14) | 6.59 (1.15) | 18.12 (6.86) | 8.96 (1.03) | 10.7 (1.15) | 7.23 (0.44) | 13.4 (1.22) |
| TSH (mU/L) | 1.24 (0.61) | 11.71 (14.19) | 0.05 (0.03) | 4.42 (2.06) | 0.07 (0.02) | 1.46 (0.69) | 1.1 (0.7) |
| Anti-TPO Positive n (%) | 70 (4.25%) | 9 (69.23%) | 4 (33.33%) | 22 (39.29%) | 1 (3.23%) | 4 (11.11%) | 4 (14.29%) |

## Overt hypothyroidism (n=13)

| Neurodevelopmental outcome | Unadjusted model | | | Adjusted model | | | | |
| --- | --- | --- | --- | --- | --- | --- | --- | --- |
|  | **estimate** | **std error** | **p value** | **estimate** | **std error** | **p value** | **lower CI** | **upper CI** |
| Non-verbal intelligence | 0.06 | 0.14 | 0.65 | 0.12 | 0.14 | 0.41 | -0.16 | 0.4 |
| Executive working memory | -0.45 | 0.26 | 0.09 | -0.49 | 0.28 | 0.08 | -1.05 | 0.06 |
| Behavioural Regulation | -0.08 | 0.1 | 0.47 | -0.06 | 0.1 | 0.55 | -0.27 | 0.14 |
| Metacognition | -0.06 | 0.08 | 0.48 | -0.06 | 0.08 | 0.49 | -0.22 | 0.1 |
| Internalising behaviour | -0.21 | 0.13 | 0.12 | -0.22 | 0.13 | 0.10 | -0.47 | 0.04 |
| Risk Taking Behaviour | -0.11 | 0.17 | 0.51 | -0.09 | 0.17 | 0.59 | -0.42 | 0.24 |
| Mother-Reported Externalising Problems | -0.23 | 0.35 | 0.51 | -0.18 | 0.34 | 0.60 | -0.85 | 0.49 |
| Mother-Reported Internalising Problems | 0.24 | 0.35 | 0.5 | 0.28 | 0.35 | 0.42 | -0.4 | 0.96 |
| Teacher-Reported Externalising Problems | 0.2 | 0.41 | 0.63 | -0.06 | 0.42 | 0.88 | -0.89 | 0.76 |
| Teacher-Reported Internalising Problems | 0.64 | 0.34 | 0.06 | 0.35 | 0.37 | 0.34 | -0.38 | 1.08 |
| Self-Reported Externalising Problems | -0.29 | 0.23 | 0.22 | -0.28 | 0.23 | 0.22 | -0.73 | 0.17 |
| Self-Reported Internalising Problems | 0.13 | 0.27 | 0.62 | 0.14 | 0.26 | 0.58 | -0.37 | 0.66 |

## Overt hyperthyroidism (n=12)

| Neurodevelopmental outcome | Unadjusted model | | | Adjusted model | | | | |
| --- | --- | --- | --- | --- | --- | --- | --- | --- |
|  | **estimate** | **std error** | **p value** | **estimate** | **std error** | **p value** | **lower CI** | **upper CI** |
| Non-verbal intelligence | -0.06 | 0.16 | 0.7 | -0.06 | 0.16 | 0.70 | -0.38 | 0.25 |
| Executive working memory | -0.05 | 0.36 | 0.9 | 0.01 | 0.36 | 0.98 | -0.69 | 0.71 |
| Behavioural Regulation | -0.03 | 0.09 | 0.77 | -0.02 | 0.09 | 0.85 | -0.19 | 0.15 |
| Metacognition | -0.04 | 0.07 | 0.59 | -0.02 | 0.07 | 0.80 | -0.16 | 0.12 |
| Internalising behaviour | 0.23 | 0.11 | 0.04 | 0.17 | 0.12 | 0.15 | -0.06 | 0.4 |
| Risk Taking Behaviour | 0.31 | 0.15 | 0.03 | 0.24 | 0.15 | 0.11 | -0.05 | 0.54 |
| Mother-Reported Externalising Problems | -0.28 | 0.3 | 0.36 | -0.22 | 0.3 | 0.46 | -0.8 | 0.36 |
| Mother-Reported Internalising Problems | -0.01 | 0.31 | 0.98 | -0.01 | 0.31 | 0.97 | -0.62 | 0.59 |
| Teacher-Reported Externalising Problems | -0.42 | 0.55 | 0.45 | -0.13 | 0.52 | 0.80 | -1.15 | 0.88 |
| Teacher-Reported Internalising Problems | -0.39 | 0.51 | 0.44 | -0.41 | 0.51 | 0.42 | -1.41 | 0.59 |
| Self-Reported Externalising Problems | 0.14 | 0.2 | 0.49 | 0.07 | 0.21 | 0.72 | -0.34 | 0.49 |
| Self-Reported Internalising Problems | 0.09 | 0.26 | 0.72 | -0.01 | 0.27 | 0.96 | -0.54 | 0.51 |

## Subclinical Hypothyroidism (n=56)

| Neurodevelopmental outcome | Unadjusted model | | | Adjusted model | | | | |
| --- | --- | --- | --- | --- | --- | --- | --- | --- |
|  | **estimate** | **std error** | **p value** | **estimate** | **std error** | **p value** | **lower CI** | **upper CI** |
| Non-verbal intelligence | 0.1 | 0.06 | 0.11 | 0.09 | 0.06 | 0.14 | -0.03 | 0.22 |
| Executive working memory | 0.11 | 0.13 | 0.39 | 0.13 | 0.13 | 0.31 | -0.12 | 0.37 |
| Behavioural Regulation | 0.01 | 0.04 | 0.84 | 0.01 | 0.04 | 0.77 | -0.07 | 0.1 |
| Metacognition | 0.03 | 0.04 | 0.34 | 0.03 | 0.03 | 0.33 | -0.03 | 0.1 |
| Internalising behaviour | -0.04 | 0.06 | 0.51 | -0.04 | 0.06 | 0.48 | -0.15 | 0.07 |
| Risk Taking Behaviour | 0.05 | 0.08 | 0.52 | 0.05 | 0.07 | 0.53 | -0.1 | 0.19 |
| Mother-Reported Externalising Problems | 0.1 | 0.14 | 0.5 | 0.11 | 0.14 | 0.43 | -0.16 | 0.38 |
| Mother-Reported Internalising Problems | -0.07 | 0.16 | 0.64 | -0.1 | 0.16 | 0.53 | -0.41 | 0.21 |
| Teacher-Reported Externalising Problems | 0.1 | 0.22 | 0.65 | 0.2 | 0.21 | 0.35 | -0.22 | 0.62 |
| Teacher-Reported Internalising Problems | -0.14 | 0.21 | 0.51 | -0.12 | 0.21 | 0.57 | -0.53 | 0.29 |
| Self-Reported Externalising Problems | 0.03 | 0.1 | 0.74 | 0.03 | 0.09 | 0.71 | -0.15 | 0.22 |
| Self-Reported Internalising Problems | -0.12 | 0.12 | 0.33 | -0.12 | 0.12 | 0.34 | -0.36 | 0.12 |

## Subclinical Hyperthyroidism (n=31)

| Neurodevelopmental outcome | Unadjusted model | | | Adjusted model | | | | |
| --- | --- | --- | --- | --- | --- | --- | --- | --- |
|  | **estimate** | **std error** | **p value** | **estimate** | **std error** | **p value** | **lower CI** | **upper CI** |
| Non-verbal intelligence | -0.14 | 0.14 | 0.30 | -0.2 | 0.14 | 0.16 | -0.48 | 0.08 |
| Executive working memory | 0.17 | 0.27 | 0.52 | 0.21 | 0.27 | 0.42 | -0.31 | 0.73 |
| Behavioural Regulation | -0.08 | 0.06 | 0.23 | -0.07 | 0.06 | 0.24 | -0.2 | 0.05 |
| Metacognition | -0.05 | 0.05 | 0.35 | -0.02 | 0.05 | 0.74 | -0.11 | 0.08 |
| Internalising behaviour | -0.02 | 0.08 | 0.84 | -0.04 | 0.08 | 0.64 | -0.19 | 0.12 |
| Risk Taking Behaviour | -0.04 | 0.1 | 0.69 | 0.00 | 0.1 | 0.97 | -0.2 | 0.19 |
| Mother-Reported Externalising Problems | **-0.55** | **0.23** | **0.02** | **-0.46** | **0.22** | **0.04** | **-0.9** | **-0.03** |
| Mother-Reported Internalising Problems | -0.25 | 0.23 | 0.28 | -0.26 | 0.23 | 0.25 | -0.72 | 0.19 |
| Teacher-Reported Externalising Problems | -0.87 | 0.56 | 0.12 | -0.39 | 0.53 | 0.46 | -1.44 | 0.65 |
| Teacher-Reported Internalising Problems | -0.24 | 0.46 | 0.6 | -0.19 | 0.46 | 0.68 | -1.08 | 0.71 |
| Self-Reported Externalising Problems | -0.09 | 0.13 | 0.51 | -0.07 | 0.13 | 0.59 | -0.33 | 0.19 |
| Self-Reported Internalising Problems | -0.12 | 0.17 | 0.49 | -0.21 | 0.17 | 0.22 | -0.54 | 0.13 |

## Clinical Hypothyroxinaemia (n=36)

| Neurodevelopmental outcome | Unadjusted model | | | Adjusted model | | | | |
| --- | --- | --- | --- | --- | --- | --- | --- | --- |
|  | **estimate** | **std error** | **p value** | **estimate** | **std error** | **p value** | **lower CI** | **upper CI** |
| Non-verbal intelligence | -0.08 | 0.09 | 0.39 | -0.06 | 0.09 | 0.51 | -0.24 | 0.12 |
| Executive working memory | 0.01 | 0.17 | 0.96 | -0.18 | 0.18 | 0.33 | -0.54 | 0.18 |
| Behavioural Regulation | 0.00 | 0.05 | 0.93 | -0.02 | 0.05 | 0.67 | -0.13 | 0.08 |
| Metacognition | -0.01 | 0.04 | 0.83 | -0.03 | 0.04 | 0.43 | -0.12 | 0.05 |
| Internalising behaviour | 0.13 | 0.08 | 0.09 | 0.04 | 0.08 | 0.58 | -0.11 | 0.2 |
| Risk Taking Behaviour | 0.09 | 0.1 | 0.34 | 0.02 | 0.1 | 0.83 | -0.18 | 0.22 |
| Mother-Reported Externalising Problems | 0.16 | 0.17 | 0.36 | 0.04 | 0.17 | 0.83 | -0.3 | 0.37 |
| Mother-Reported Internalising Problems | 0.34 | 0.18 | 0.06 | 0.21 | 0.18 | 0.24 | -0.15 | 0.57 |
| Teacher-Reported Externalising Problems | 0.56 | 0.28 | 0.05 | 0.53 | 0.28 | 0.06 | -0.03 | 1.08 |
| Teacher-Reported Internalising Problems | 0.58 | 0.25 | 0.02 | 0.46 | 0.26 | 0.07 | -0.04 | 0.96 |
| Self-Reported Externalising Problems | 0.08 | 0.13 | 0.51 | -0.01 | 0.13 | 0.94 | -0.27 | 0.25 |
| Self-Reported Internalising Problems | 0.11 | 0.16 | 0.5 | 0.00 | 0.16 | 0.99 | -0.32 | 0.32 |

## Clinical Hyperthyroxinaemia (n=28)

| Neurodevelopmental outcome | Unadjusted model | | | Adjusted model | | | | |
| --- | --- | --- | --- | --- | --- | --- | --- | --- |
|  | **estimate** | **std error** | **p value** | **estimate** | **std error** | **p value** | **lower CI** | **upper CI** |
| Non-verbal intelligence | -0.12 | 0.1 | 0.22 | -0.12 | 0.09 | 0.21 | -0.3 | 0.07 |
| Executive working memory | 0.2 | 0.19 | 0.29 | 0.21 | 0.19 | 0.25 | -0.15 | 0.58 |
| Behavioural Regulation | 0.02 | 0.06 | 0.72 | 0.02 | 0.06 | 0.69 | -0.09 | 0.14 |
| Metacognition | 0.02 | 0.05 | 0.73 | 0.01 | 0.05 | 0.80 | -0.08 | 0.1 |
| Internalising behaviour | -0.1 | 0.08 | 0.18 | -0.1 | 0.08 | 0.21 | -0.26 | 0.06 |
| Risk Taking Behaviour | 0.06 | 0.1 | 0.57 | 0.03 | 0.1 | 0.75 | -0.17 | 0.24 |
| Mother-Reported Externalising Problems | 0.11 | 0.19 | 0.57 | 0.07 | 0.19 | 0.71 | -0.3 | 0.44 |
| Mother-Reported Internalising Problems | 0 | 0.21 | 1.00 | 0.06 | 0.21 | 0.78 | -0.35 | 0.47 |
| Teacher-Reported Externalising Problems | 0.29 | 0.29 | 0.32 | 0.16 | 0.28 | 0.56 | -0.38 | 0.71 |
| Teacher-Reported Internalising Problems | -0.13 | 0.27 | 0.64 | -0.13 | 0.28 | 0.64 | -0.68 | 0.42 |
| Self-Reported Externalising Problems | -0.15 | 0.14 | 0.28 | -0.16 | 0.15 | 0.27 | -0.46 | 0.13 |
| Self-Reported Internalising Problems | **-0.48** | **0.2** | **0.01** | **-0.43** | **0.21** | **0.04** | **-0.83** | **-0.03** |
