# Supplementary material for: Maternal thyroid function in the first half of pregnancy and neurodevelopmental outcomes in early adolescence in the Amsterdam Born Children and their Development (ABCD) cohort
Source: Compr Psychoneuroendocrinol. 2025 Dec 22;25:100333. doi: 10.1016/j.cpnec.2025.100333 (PMC12808570; doi:10.1016/j.cpnec.2025.100333)
Supplement: Multimedia component 8 [file mmc8.docx]

Supplementary 8

# Extensive information regarding the comparison between anti-TPO positive and negative mother-child dyads

| Variable | negative | positive |
| --- | --- | --- |
| Number of Mother-Child Pairs | 1710 | 114 |
| Percentage of girls | 881 (51.52%) | 63 (55.26%) |
| Age of Child At Testing | 11.56 (0.31) | 11.59 (0.3) |
| Maternal Age During Pregnancy | 32.02 (4.1) | 32.26 (3.65) |
| Parity | 0.52 (0.75) | 0.57 (0.78) |
| Percentage Dutch ethnicity | 1258 (73.57%) | 76 (66.67%) |
| Maternal Education Years | 10.27 (3.41) | 10.68 (3.26) |
| Percentage Smoking | 116 (6.78%) | 5 (4.39%) |
| Pre-Pregnancy BMI | 22.67 (3.44) | 22.64 (3.1) |
| Birth Weight Child | 3507.06 (538.99) | 3514.31 (610.1) |
| Percentage Gestational Diabetes | 12.93 (2.13) | 12.95 (2.04) |
| Percentage Gestational Hypertension | 9.76 (1.4) | 9.41 (3.1) |
| Thyroid Testing Gestational Week | 1.26 (0.78) | 3.24 (5.85) |
| FT4 | 0 (0%) | 114 (100%) |
| FastTSH | 1710 | 114 |
| Percentage Anti-TPO Positive | 881 (51.52%) | 63 (55.26%) |

| Neurodevelopmental outcome | Unadjusted model | | | Adjusted model | | | | |
| --- | --- | --- | --- | --- | --- | --- | --- | --- |
|  | **estimate** | **std error** | **p value** | **estimate** | **std error** | **p value** | **lower CI** | **upper CI** |
| Non-verbal intelligence | 0.06 | 0.05 | 0.19 | 0.07 | 0.05 | 0.16 | -0.03 | 0.17 |
| Executive working memory | -0.15 | 0.1 | 0.13 | -0.12 | 0.1 | 0.24 | -0.31 | 0.08 |
| Behavioural Regulation | 0.02 | 0.03 | 0.58 | 0.02 | 0.03 | 0.52 | -0.04 | 0.08 |
| Metacognition | 0 | 0.03 | 0.94 | 0.02 | 0.02 | 0.54 | -0.03 | 0.06 |
| Internalising behaviour | 0.03 | 0.04 | 0.48 | 0.01 | 0.04 | 0.82 | -0.07 | 0.09 |
| Risk Taking Behaviour | 0.07 | 0.05 | 0.21 | 0.08 | 0.05 | 0.13 | -0.02 | 0.19 |
| Mother-Reported Externalising Problems | 0.02 | 0.1 | 0.83 | 0.09 | 0.1 | 0.37 | -0.11 | 0.29 |
| Mother-Reported Internalising Problems | 0.11 | 0.11 | 0.33 | 0.12 | 0.11 | 0.28 | -0.09 | 0.33 |
| Teacher-Reported Externalising Problems | 0 | 0.16 | 0.99 | 0.23 | 0.16 | 0.14 | -0.08 | 0.54 |
| Teacher-Reported Internalising Problems | 0.02 | 0.14 | 0.88 | 0.02 | 0.15 | 0.89 | -0.27 | 0.31 |
| Self-Reported Externalising Problems | 0 | 0.07 | 1 | 0.01 | 0.07 | 0.85 | -0.13 | 0.15 |
| Self-Reported Internalising Problems | 0.04 | 0.09 | 0.65 | 0.01 | 0.09 | 0.93 | -0.17 | 0.19 |
